# Expression of the Biologically Active Insulin Analog SCI-57 in *Nicotiana Benthamiana*

**DOI:** 10.3389/fphar.2019.01335

**Published:** 2019-11-14

**Authors:** Adriana Muñoz-Talavera, Miguel Ángel Gómez-Lim, Luis A. Salazar-Olivo, Jörg Reinders, Katharina Lim, Abraham Escobedo-Moratilla, Alberto Cristian López-Calleja, María Cristina Islas-Carbajal, Ana Rosa Rincón-Sánchez

**Affiliations:** ^1^Department of Physiology, Institute of Experimental and Clinical Therapeutics, University Center for Health Sciences, University of Guadalajara, Guadalajara, Mexico; ^2^Department of Genetic Engineering, Center for Research and Advanced Studies of the National Polytechnic Institute, Irapuato, Mexico; ^3^Division of Molecular Biology, Institute for Scientific and Technological Research of San Luis Potosí, San Luis Potosí, Mexico; ^4^Scientific Support Unit Analytical Chemistry, Leibniz Research Centre for Working Environment and Human Factors, Dortmund, Germany; ^5^Institute of Functional Genomics, University of Regensburg, Regensburg, Germany; ^6^CONACYT-Consortium for Research, Innovation, and Development of the Drylands (CIIDZA), IPICYT, San Luis Potosí, Mexico; ^7^Institute of Molecular Biology and Gene Therapy, Department of Molecular Biology and Genomic, University Center for Health Sciences, University of Guadalajara, Guadalajara, Mexico

**Keywords:** diabetes, insulin analog SCI-57, proteomic profile, *Nicotiana benthamiana*, transient expression, 3T3-L1 adipocytes

## Abstract

Diabetes mellitus is a growing problem worldwide; however, only 23% of low-income countries have access to insulin, and ironically it costs higher in such countries than high-income ones. Therefore, new strategies for insulin and insulin analogs production are urgently required to improve low-cost access to therapeutic products, so as to contain the diabetes epidemic. SCI-57 is an insulin analog with a greater affinity for the insulin receptor and lower thermal degradation than native insulin. It also shows native mitogenicity and insulin-like biological activity. In this work, SCI-57 was transiently expressed in the *Nicotiana benthamiana* (*Nb*) plant, and we also evaluated some of its relevant biological effects. An expression plasmid was engineered to translate an N-terminal ubiquitin and C-terminal endoplasmic reticulum-targeting signal KDEL, in order to increase protein expression and stability. Likewise, the effect of co-expression of influenza M2 ion channel (M2) on the expression of insulin analog SCI-57 (SCI-57/M2) was evaluated. Although using M2 increases yield, it tends to alter the SCI-57 amino acid sequence, possibly promoting the formation of oligomers. Purification of SCI-57 was achieved by FPLC cation exchange and ultrafiltration of *N. benthamiana* leaf extract (NLE). SCI-57 exerts its anti-diabetic properties by stimulating glucose uptake in adipocytes, without affecting the lipid accumulation process. Expression of the insulin analog in agroinfiltrated plants was confirmed by SDS-PAGE, RP-HPLC, and MS. Proteome changes related to the expression of heterologous proteins on *N. benthamiana* were not observed; up-regulated proteins were related to the agroinfiltration process. Our results demonstrate the potential for producing a biologically active insulin analog, SCI-57, by transient expression in *Nb*.

## Introduction

Diabetes is a public health problem, as it has been estimated that the number of patients with diabetes in 2040 will be 642 million, representing 10% of world population (Ogurtsova et al., 2017).

In order to manage the autoimmune destruction of insulin-producing pancreatic beta cells in type 1 diabetes (DM1), it is necessary to administer insulin *via* lifelong daily injection. Currently, worldwide, DM1 represents 5% to 10% of all diabetes cases (You and Henneberg, 2016), however, the use of insulin is not limited to only those people afflicted with DM1. A prospective study on diabetes in the UK (UKPDS) showed that to achieve better glycemic control, after five years, more than 50% of people with type 2 diabetes (DM2) required additional medication such as insulin (King et al., 1999). Similarly, general recommendations for patients with DM2, published by the American Diabetes Association (ADA) and the European Association for the Study of Diabetes (EASD), include insulin and insulin analogs as treatment options in dual and triple anti-hyperglycemic therapy (Inzucchi et al., 2015).

As a consequence of the increasing incidence of diabetes, the need for insulin and insulin analogs will augment exponentially. Therefore, new strategies for insulin and insulin analog production are urgently required, in order to increase the availability of therapeutic products for containing the diabetes epidemic. Likewise, globally, only 23% of low-income countries reported that insulin is generally available, although at higher costs than high-income countries (WHO, 2016). The average insulin cost increased three-fold from 2002 to 2013, with availability and rising costs having an impact on diabetes patients and health systems worldwide (Hua et al., 2016). For these reasons, the study of new platforms for the expression of insulin and insulin analogs costs is of utmost importance.

Recombinant human insulin is predominantly produced using *Escherichia coli* and yeast. *E. coli*, as expression system implicates several disadvantages; insulin is obtained *via* the production of insulin precursors (IP), therefore *in vitro* cleavage and oxidative refolding are necessary. In addition, some IP forms inclusion bodies requiring solubilization. With regard to yeast-based expression systems, although the insulin is correctly folded and directly secreted in the culture supernatant, the standard recovery and purification process may require numerous steps (Mollerup et al., 2002).

Plants are considered to be safe, effective, and affordable alternative systems for producing a wide variety of recombinant proteins such as enzymes, vaccines, and other biopharmaceuticals (Ma et al., 2005; Redwan, 2007; Castillo-Esparza, 2014). Their most important advantage over bacterial, yeast or mammal systems relates to the low cost of large-scale production (Twyman et al., 2003; Ma et al., 2005), partly because the necessary processes comprise existing agricultural systems, thus reducing operating and capital costs (Lico et al., 2008). Studies carried out by Tuse and collaborators have shown that costs can be as high as 1.00–2.00 dollars per kilogram of protein (Tuse et al., 2014). Plant-based expression systems hold tremendous potential for high-capacity production of insulin, in a very cost-effective manner, and may also contend with bacterial and yeast disadvantages such as extensive purification requirements (Baeshen et al., 2014). Recombinant human insulin has been successfully produced in transgenic *Arabidopsis thaliana* (Nykiforuk et al., 2006), and human proinsulin in transgenic tobacco and lettuce chloroplasts (Boyhan and Daniell, 2011), creating a natural cell store with long-term stability that provides storage until required.

Recombinant proteins can be produced in plants by genetic transformation or transient expression. Stable transformation involves the chromosomal integration of the gene of interest. This process can therefore take substantial time (often years) and require considerable resources. Likewise, the expressed protein yield is relatively low, and the release of a transgenic plant implies biosecurity problems (Edelbaum et al., 1992; Kusnadi et al., 1997). Recent studies have shown that proteins generated by transient expression (magnifection) have benefits, as human pathogens are not involved; thus, sterility is not required during production, meaning expression is rapid and high-level, with the potential to provide gram quantities of product in less than 4 weeks to use in clinical trials for FDA approval (Buyel, 2018). Phase I of the clinical study to produce immunoglobulins for the treatment of non-Hodgkin’s lymphoma, by transient expression in *Nicotiana benthaminana*, has been completed (Klimyuk et al., 2014). Another recent development in this field is the production of ZMapp, a combination of three chimeric monoclonal antibodies destined to the treatment of Ebola virus disease. This combination is being developed as a product of molecular agriculture by the Leaf Biopharmaceutical Company and is produced by transient expression in *N. benthamiana* (Budzianowski, 2015; Hiatt et al., 2015). ZMapp was approved by the FDA and WHO during the Ebola outbreak in West Africa in 2014, because the transient expression system allowed rapid production, and previous studies had shown positive results among primates (Qiu et al., 2014). Subsequently, ZMapp began formal clinical development and recently completed phase II trials in Liberia, Sierra Leone, Guinea, and the United States (Na et al., 2015; Davey et al., 2016).

SCI-57 is an insulin analog that has a ten-fold affinity for the insulin receptor, higher resistance to thermal degradation than insulin, native mitogenicity and biological effect. The objective of this work was to transiently express the insulin analog SCI-57 in *N. benthamiana* leaves, purify, and characterize the protein by Reversed-Phase High-Performance Liquid Chromatography (RP-HPLC), mass spectrometry (MS), Diagonal two-dimensional electrophoresis (D-2DE) and enzyme-linked immunosorbent assay (ELISA). Here, we developed a new strategy for the production and purification of SCI-57 in plant leaves, evaluating some of its diabetes-relevant biological effects such as triglyceride accumulation and 2-NBDG uptake in 3T3-L1 adipocytes.

## Materials and Methods

### Construction of Expression Vectors

The native version of the protein was obtained from the Protein Data Base (PDBID: 2JZQ). Three molecular strategies were used to increase the expression and accumulation of the insulin analog SCI-57: 1) The addition of a retention signal (KDEL peptide) at the carboxyl-terminus end to direct the recombinant protein to the endoplasmic reticulum, ensuring its correct folding and accumulation (Fischer and Emans, 2000; Cabrera et al., 2003; Conley et al., 2011; Giritch et al., 2015; Lacombe et al., 2018). 2) Ubiquitin fusion at amino-terminus to increase protein expression levels (Hondred et al., 1999; Streatfield, 2007; Benchabane et al., 2008); the ubiquitin at the plasmid construct was located on the 5’ end, downstream of the translationally silent *BsaI* site, and 10 bp from the start codon (**Supplementary Figure S1**). 3) Co-expression of the ion channel of the influenza virus (M2) to increase the yield and quality of the recombinant protein (Sainsbury et al., 2013; Jutras et al., 2015); M2 was on the pMDC85 plasmid (PMDC85-M2). For optimal expression in plants, the gene encoding SCI-57 was codon optimized and synthesized (GenScript, USA). The 451 bp fragment corresponding to SCI-57 gene, KDEL peptide, and ubiquitin was digested with *Bsa*I and subcloned in the *Bsa*I site of pICH31070, in order to obtain the pICH31070-SCI-57 expression vector. pICH31070 containing green fluorescent protein (GFP) was used as positive control (pICH31070-GFP).

The vectors were introduced—the 5′ module (pICH15879), the 3′ module (pICH31070-SCI-57, pICH31070-GFP, PMDC85-M2), and the integrase module (pICH14011)—in *Agrobacterium tumefaciens* GV3101 strain by electroporation.

### Agroinfiltration of *N. Benthamiana* Plants

The infiltration procedure was carried out, following our laboratory procedure (Coconi-Linares et al., 2013). In brief, GV3101 cells were incubated at 28°C with constant agitation (180 rpm) in YEB liquid medium with 50 mg/l kanamycin until the OD 600_nm_ = 1.0. Subsequently, cultures were diluted with infiltration buffer (10 mM MES (4-morpholineethanesulfonic acid) pH 5.5, 10 mM MgSO_4_ and 100 µM acetosyringone) to achieve an OD 600_nm_ = 0.6.

The bacterial suspension containing pICH31070-GFP was mixed with equal volumes of the 5′ module and integrase module suspension. The bacterial suspension containing pICH31070-SCI-57 was mixed with equal volumes of the 5′ module and integrase module suspension, with or without pMDC85-M2 suspension to evaluate the M2 role in SCI-57 expression.

**Figure 1:**
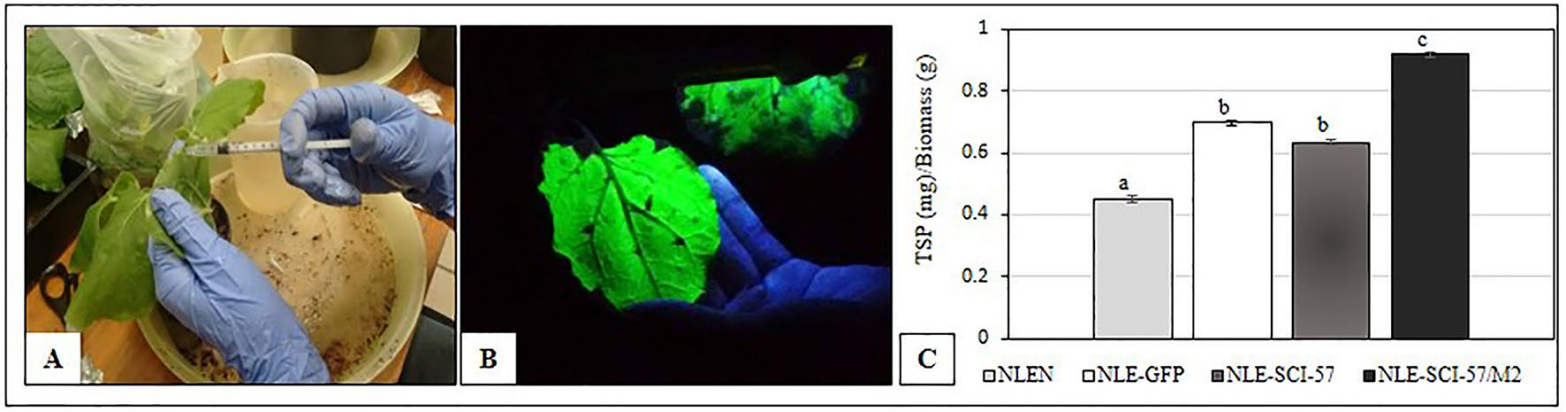
**(A)** Syringe agroinfiltration of *Nicotiana benthamiana* leaves with *Agrobacterium tumefaciens*. **(B)** Transient expression of GFP in leaves of *N. benthamiana* 4 days after the agroinfiltration. **(C)** Bradford analysis of the total soluble proteins (TSP). Leaves were harvested four days post-agroinfiltration. Results are presented as the mean ± S.D. of three independent experiments in triplicate. Lowercase letters indicate significant differences according to the Mann-Whitney test (*p* ≤ 0.05). NLEN, *N. benthamiana*-leaf extract from non-agroinfiltrated plants; NLE-GFP, *N. benthamiana*-leaf extract from GFP expressing plants; NLE-SCI-57, *N. benthamiana*-leaf extract from SCI-57 expressing plants; NLE-SCI-57/M2, *N. benthamiana*-leaf extract from SCI-57 expressing plants and co-expressing M2.

Bacterial suspension mixtures were infiltrated into plant leaves using syringes (**Figure 1A**). After infiltration, the plants were grown at 25°C during a 16-hour light/8-hour dark photoperiod, until GFP expression was observed over most of the leaf surface area (**Figure 1B**). After 4–5 days, the leaves were harvested and macerated using a mortar and pestle, in the presence of liquid nitrogen and stored at −80°C.

### Extraction of Total Soluble Proteins (TSP) and SDS-PAGE

The macerated tissue was mixed and homogenized with the extraction buffer: 100 mM citrate buffer pH 4.3, 1 µl of protease inhibitor cocktail (Sigma-Aldrich, USA), and 10 µl of cellulases per ml (Roche, USA) at a ratio of 1 g leaf tissue to 1.5 ml buffer. Subsequently, this was incubated at 37°C for 1 h and afterwards centrifuged at 10,000 rpm for 15 min at 4°C. The resulting solution was filtered with cellulose acetate membranes of 0.8, 0.45, and 0.22 µm (Millipore Corporation, USA) in order to remove solids and pigments. The NLE was stored at 4°C for subsequent analysis.

We used a low pH extraction buffer to precipitate contaminants such as cell debris and photosynthetic pigments present in green tissues like the leaves of *N. benthamiana*. In addition, this method was also useful for precipitating the protein ribulose-1,5-bisphosphate carboxylase/oxygenase (RuBisCO) which represents approximately 50% of the TSP present in the leaves and usually complicates the generation of highly pure recombinant proteins (Bendandi et al., 2010; Lai et al., 2010; Tuse et al., 2015).

In order to measure the TSP in NLE, we conducted the Bradford analysis. A standard protein curve was constructed using bovine serum albumin (Sigma-Aldrich, USA) from 0 to 1.5 mg/ml, using the extraction buffer as diluent. The absorbance of the standards and samples was measured at 595 nm.

TSP were separated by 13% Tricine–SDS–PAGE under reducing conditions (5% β-mercaptoethanol) and samples were heated for 5 min at 95°C. Electrophoresis was performed for 90 min at 120V. Gels were visualized with Coomassie blue stain (Sigma-Aldrich, USA).

### Extraction of Total RNA and cDNA Preparation

Total RNA was extracted from 100–200 mg of frozen agroinfiltrated leaves, following the manufacturer´s procedure for TRIzol (Invitrogen, USA). The quality of these samples was evaluated by the presence of ribosomal bands on the ethidium bromide-stained agarose gel electrophoresis. Accordingly, total RNA was treated with DNase (Invitrogen, USA). RNA samples (5 µg) were reverse transcribed to generate single-stranded cDNA using an oligo (dT)18 Primer and 200 units of SuperScript III reverse transcriptase, as described by the manufacturer (Invitrogen, USA).

### Gene Analysis by Endpoint PCR and Quantitative Real-Time PCR (qRT-PCR)

Primers were designed using the Primer select software (Graham and Holland, 2005) and validated with the help of the OligoAnalyzer program (Owczarzy et al., 2008) (data available in **Supplementary Table S1**). They were synthesized by the T4 Oligo company (T4 Oligo, México). Endpoint PCR was performed to amplify 18S rRNA, GFP and SCI-57, whereas qRT-PCR assays were performed to amplify 18S rRNA and SCI-57.

End-point PCR samples were amplified by applying the following program: initial denaturation at 98°C for 3 min, followed by 30 cycles of denaturation for 45 s at 94°C, annealing for 30 s at 60.4°C, and elongating for 30 s at 72°C. End-point PCR assays were performed on MultiGene^™^ OptiMax Thermal Cycler (Labnet International Inc., USA), and the enzyme Taq polymerase was used as described by the manufacturer (Invitrogen, USA) for a reaction volume of 25 µl with 250 ng of cDNA. No template controls (NTCs) were included with each instrument run for quality control, and 18S rRNA was chosen as the housekeeping gene. Samples were analyzed by 2% ethidium bromide-stained agarose gel electrophoresis.

qRT-PCR samples were diluted to a 100 ng/µl single-stranded cDNA concentration with sterile water. The CFX96 real-time PCR detection system (Bio-Rad, USA) was used for all qRT-PCRs. Samples were amplified using SYBR Green with the following program: initial denaturation at 95°C for 3 min, followed by 40 cycles of denaturation for 5 s at 98°C, annealing for 30 s at 60.4°C, and elongating for 30 s at 72°C. Amplification reactions were prepared using a total volume of 10 µl. PCR was followed by a standard melting curve analysis. All PCRs were run in triplicate, and control reactions without template were included in each assay. The 18S rRNA gene was used as a reference for normalization. The data was analyzed using the Bio-Rad CFX Manager 3.1 management software. Relative expression was determined by the evaluation of the expression by the 2^−ΔΔCT^ (Livak and Schmittgen, 2001) method.

### Purification of SCI-57

#### Cation Exchange Fast Protein Liquid Chromatography (FPLC)

Analysis was carried out with a column of SP-Sepharose Fast Flow (GE Healthcare, Sweden) connected to the FPLC equipment (ÄKTA avant 25, Amersham Biosciences, USA). Recombinant human insulin (PISA pharmaceutical, México) on citrate buffer was used as positive control (1.75 mg/ml). The column was equilibrated with 10 ml of binding buffer (100 mM citrate buffer pH 4.3). Then, 5 ml of NLE or insulin solution was passed through the column. The column was washed with 5 ml of washing buffer (100 mM citrate buffer pH 4.3). The mobile phases were buffer A (100 mM citrate buffer pH 4.3) and buffer B (100 mM citrate buffer pH 4.3 with 1M NaCl). The elution profile to achieve the separation constituted a gradient of 100% B for 60 min with a continuous flow rate of 0.4 ml/min; samples were collected in 1 ml fractions. Elution fractions were monitored by a single path ultraviolet monitor at 280 nm. SCI-57 presence on the fractions was evaluated by 13% Tricine SDS-PAGE.

The fractions containing SCI-57 were filtrated through centrifugal filter devices with a cutoff of 3 kDa (Millipore, USA).

### Reversed-Phase High-Performance Liquid Chromatography (RP-HPLC) Analysis

Samples were analyzed using the HPLC System Agilent 12900 Infinity II (Agilent Technologies, USA). The diode array detector was set to collect signals within the spectral range of 200–400 nm. Chromatographic separation was performed on the chromatographic column (15 cm × 0.21 cm) Vydac 218MS C18 with a 5µ particle size and 300 A° pore size (Vydac, Hesperia, USA). The column was kept at 30°C in a column oven. During chromatographic separation, the mobile phase was in gradient elution from 100% A: H_2_O 0.1% trifluoroacetic acid (TFA) to 100% B: acetonitrile (ACN) 0.1% TFA over 1 h at a flow rate of 0.5 ml/min. The sample injection volume was 20 µL. Agilent ChemStation software was used for collecting and processing the data.

### Ionization Pattern and Exact Molecular Weight Determination

#### SCI-57

A 30 µM purified SCI-57 sample obtained from A2–A7 fraction concentration and solvent exchange into formic acid 0.1% by 3 kDa filter was analyzed using the SYNAPT-HDMS system (Waters Corp., USA) with ESI-Lockspray interphase. Prior to the experiments, the system was calibrated with a sodium iodide standard solution (Waters Corp., USA), and the European Pharmacopoeia Reference standard insulin (EP insulin) sample was analyzed as a positive control (30 µM).

In the experiment, data was acquired by injecting the protein solution directly into the interphase. We used a scan interval of 50–2,000 m/z with an ionizing spray voltage of 3.2 kV in positive ionization mode and a desolvation temperature of 110°C to acquire data. Molecular weight estimations were generated by the UniDec GUI versión 1.1.10 (Marty et al., 2015).

#### SCI-57 M2

The A2–A4 and A5–A7 fractions from SCI-57/M2 purification were concentrated by ultrafiltration with 3 kDa membranes and subsequently lyophilized. The resulting sample was dissolved in 0.1% TFA (16 µM), and the NaCl content was removed using C18 Zip-Tips (Millipore Corp., USA). The EP insulin standard (18 µM) was used as a positive control. Hence, the samples were dissolved in alpha-cyano-4-hydroxycinnamic acid (3.5 mg/ml in 50% acetonitrile, 0.1% TFA) and left to dry at room temperature. Molecular weight analysis was performed in the MALDI TOF/TOF Analyzer 4800 plus mass spectrophotometer (Applied Biosystems/MDS SCIEX, USA) in linear mode and with a laser intensity of 3,800 Hz.

### Identification of SCI-57 and Proteomic Profile by Gel-Assisted Sample Preparation (GASP), Using Liquid Chromatography-Tandem Mass Spectrometry (LC-MS/MS) and Applying Sequential Windowed Acquisition of All Theoretical Fragment Ion Mass Spectra (SWATH)

We used the FluoroProfile protein quantification kit to determine concentration of samples (Sigma-Aldrich Corporation), with BSA as standard. Equal amounts of protein (50 µg) were prepared in compliance with the GASP protocol, which was published by Fischer and Kessler (2015). The sample was dissolved on GASP buffer (8M urea, 2M thiourea, and 0.1 M DTT). Acrylamide/bis-acrylamide solution was added to reach a final concentration of 20%. The gel was formed by the TEMED and ammonium persulfate addition. Gel pieces were obtained by centrifugation through a plastic mesh and then fixed overnight by adding ethanol/acetic acid/water (40/10/50). In order to dehydrate the gel pieces, 1 ml of acetonitrile (ACN) was added, and then to rehydrate gel pieces, 0.5 ml 50 mM ammonium carbonate was added and shaken for 10 min. Gel pieces were dried in the speedvac for 2 h at 30°C. Trypsin solution 1:40 in 50 mM ammonium carbonate was added to the gel pieces at 37°C overnight for proteolysis. The addition of ACN and 5% formic acid were subsequently used to facilitate peptide extraction. The supernatant was dried using the speedvac and after the sample was resuspended in 5% formic acid.

From the sample containing the digested proteins, 8 µl were injected into the NanoLC 425 (AB Sciex) for peptide separation. Peptides were then separated on the YMC-Triart C18 1.9 µm, 3.0 × 150 mm column (YMC GmbH, Germany). The flow rate was set to 6 µL/min over 120-min multi-segment gradient: 0 min 98% A-2% B, 100 min 60% A-40% B, 102 min 20% A-80% B, 108 min 20% A-80% B, 110 min 98% A-2% B, 120 min 98% A-2% B (solvent A: 0.1% formic acid; solvent B: acetonitrile with 0.1% formic acid). The data set was generated when the fractionated peptides were transferred to the Q-TOF MS mass analyzer (TripleTOF 5600+, Sciex). The instrument was operated using positive ion with a mass resolution of ∼30,000 for TOF MS scan (m/z 400–1,000) and ∼15,000 for MS/MS; it was automatically calibrated after every two injections, using RePLiCal (Holman et al., 2016). A TOP20 method was used for the library runs, and variable SWATH-windows (60 windows from 400 to 1,000 m/z) were used for SWATH-MS-runs. The accumulation time for IDA was set to 250 ms for an MS1 scan and 50 ms per MS2 scan; the total cycle time was approximately 1.3 s. The accumulation time for SWATH was set to 50 ms for an MS1 scan and 35 ms per MS2 scan; the total cycle time was approximately 1.3 s (Reinders et al., 2016; Feist et al., 2018). The SWATH-library was built using the NCBI database, trypsin as protease, fragment ion mass tolerance of 0.055 Da, maximum one missed cleavage site, oxidation of methionine, pGlu for N-terminal Gln as variable modifications, only doubly and triply charged ions with the Protein Pilot 4.5 software (Sciex GmbH, Germany), and employing a 1% false discovery rate. In Skyline, a scoring model using the second best peaks for each peptide was employed using MProphet (Reiter et al., 2011). Furthermore, peak intensity, retention time difference, retention time difference squared, library intensity dot product, weighted peak shape, weighted co-elution, co-elution count, signal to noise ratio, and product mass error were factored in.

For the comparison in protein expression of the different *N. benthamiana* leaf extract, skyline MSstats was used. *N. benthamiana* leaf extract from non-agroinfiltrated plants (NLEN) were determined as a control group. *N. benthamiana* leaf extract from plants expressing GFP (NLE-GFP), *N. benthamiana* leaf extract from plants expressing SCI-57 (NLE-SCI-57), *N. benthamiana* leaf extract from plants expressing SCI-57, and co-expressing M2 (NLE-SCI-57/M2) were compared to the control group at a confidence level of 95%, resulting in the generation of a file for comparing protein expression levels. Data from Skyline software (3.7) were analyzed using Microsoft Excel 2016.

### Diagonal Electrophoresis for the Detection of Disulfide Bridges

SDS-PAGE Tricine gel was run under non-reducing conditions (Schagger, 2006). The strip of gel containing the protein sample was cut from the gel slab of the first-dimensional electrophoresis. Following treatment with 10 mM DTT + 50% glycerol and 50 mM iodoacetamide, the band was embedded into another polyacrylamide gel slab for the second-dimensional electrophoresis, under reducing conditions. The gel was silver stained according to Blum et al. (1987). The EP insulin (1 µg) was used as positive control.

### SCI-57 Detection by ELISA

SCI-57 concentration was assessed with the help of the Enzyme-Linked Immunosorbent Assay (ELISA) on the strength of the double binding test (Sandwich ELISA), using DRG Iso-Insulin ELISA Kit (No cat. EIA-2336) DRG Instruments GmbH (Germany). All technical procedures, described by the manufacturer, were adhered to.

### 3T3-L1 Adipogenesis

Confluent cultures of 3T3-L1 preadipocytes were induced to adipose differentiation with adipogenic medium (AM; L15 medium added with 10% (v/v) fetal bovine serum (FBS), 0.25 µM dexamethasone, 0.1 mM 3-isobutyl-1-methylxanthine, 1 µM d-biotin, 80 U/ml penicillin, 80 µg/ml streptomycin and 5 µg/ml insulin) (Herrera-Herrera et al., 2009). After three days, the cells were fed with maintenance medium (MM; L15 added with 10% (v/v) FBS, 100 nM insulin, 1 µM d-biotin, 80 U/ml penicillin and 80 µg/ml streptomycin). 3T3-L1 preadipocytes were cultured with non-adipogenic medium to act as negative controls (NAM; L15 added to 10% (v/v) FBS).

### Effect of SCI-57 on 3T3-L1 Adipogenesis

3T3-L1 preadipocytes were incubated with insulin-lacking adipogenic medium (AMI−) in the presence or the absence of 50 µl/ml NLE-SCI-57 or NLE-SCI-57/M2 or NLEN. Three days later, the cells were refed with insulin-lacking maintenance medium added to the respective NLE preparations. As a positive control, 3T3-L1 preadipocytes were incubated with insulin-containing AM and MM. Parallel experiments evaluated the synergic effects of insulin and NLE, by adding NLE preparations to insulin-containing medium.

After seven days in MM, the extent of lipid accumulation was estimated by staining intracellular triglycerides with oil red O (Ramirez-Zacarias et al., 1992).

### Effect of SCI-57 on 2-NBDG Uptake by 3T3-L1 Adipocytes

Cell monolayers of terminally differentiated 3T3-L1 adipocytes cultured on 96-well fluorescence plates were incubated for 60 min with PBS containing BSA 1 mg/ml and 80 µM of the fluorescent glucose analog 2-NBDG (Zapata-Bustos et al., 2014b) in the presence of NLE-SCI-57, NLE-SCI-57/M2, NLEN (50 µl/ml each), or FPLC purified fractions (25 µl/ml). Positive controls received 100 nM insulin or 10 pM oral hypoglycemic rosiglitazone (RGZ). Afterward, cell monolayers were washed with PBS to remove free 2-NBDG, and the fluorescence retained in the cells was measured on a Tecan-GENios (Tecan, Austria) fluorescence reader at an excitation wavelength of 460 nm and emission at 540 nm, using the Magellan 4.0 program. The values of 2-NBDG incorporation in the absence of insulin were subtracted from those obtained with 100 nM insulin to establish 100% specific 2-NBDG incorporation.

### Statistical Analysis

The data from most of the experiments was expressed as the mean ± the standard deviation for each group. The significant differences between groups were evaluated using non-parametric statistics. Kruskal–Wallis and Mann–Whitney U test were performed using SPSS Statistics version 20 (SPSS Inc., Chicago, IL, USA) where a p-value < 0.05 was considered statistically significant. Statistical analysis was applied to results from TSP, quantitative real-time PCR, intracellular triglycerides and 2-NBDG uptake.

## Results

### Expression of Recombinant Proteins in *N. Benthamiana*.

The 451 bp coding sequence for SCI-57 was cloned into the pICH31070 plasmid. The *Bgl*II restriction analysis confirmed their presence with a resulting plasmid of 5,393 bp in size, as anticipated (**Supplementary Figure S1**).

It was necessary to recombine pro-vector modules within the cell of the plant in order to produce SCI-57. It was thus essential to ascertain that pro-vector assembly was carried out correctly, and that the transient expression of recombinant proteins in *N. benthamiana* was not affected by external factors. GFP was used as a visible tool to confirm the pro-vector assembly *in vivo* and therefore the SCI-57 expression. Four days after agroinfiltration (**Figure 1A**), GFP could be observed on the majority of leaf surfaces (**Figure 1B**). Leaves were thus harvested to obtain NLE. The quantity of TSP on NLE was determined by the Bradford assay. The Bradford assay revealed a TSP concentration which was at least 1.4 times higher in NLE from plants expressing heterologous proteins compared to NLEN. The greatest amount of TSP was observed on NLE-SCI-57/M2, followed by NLE-GFP and NLE-SCI-57. The results were presented as the mean ± S.D. of three independent experimental values for TPS (mg)/biomass (g) (**Figure 1C**).

**Figure 2:**
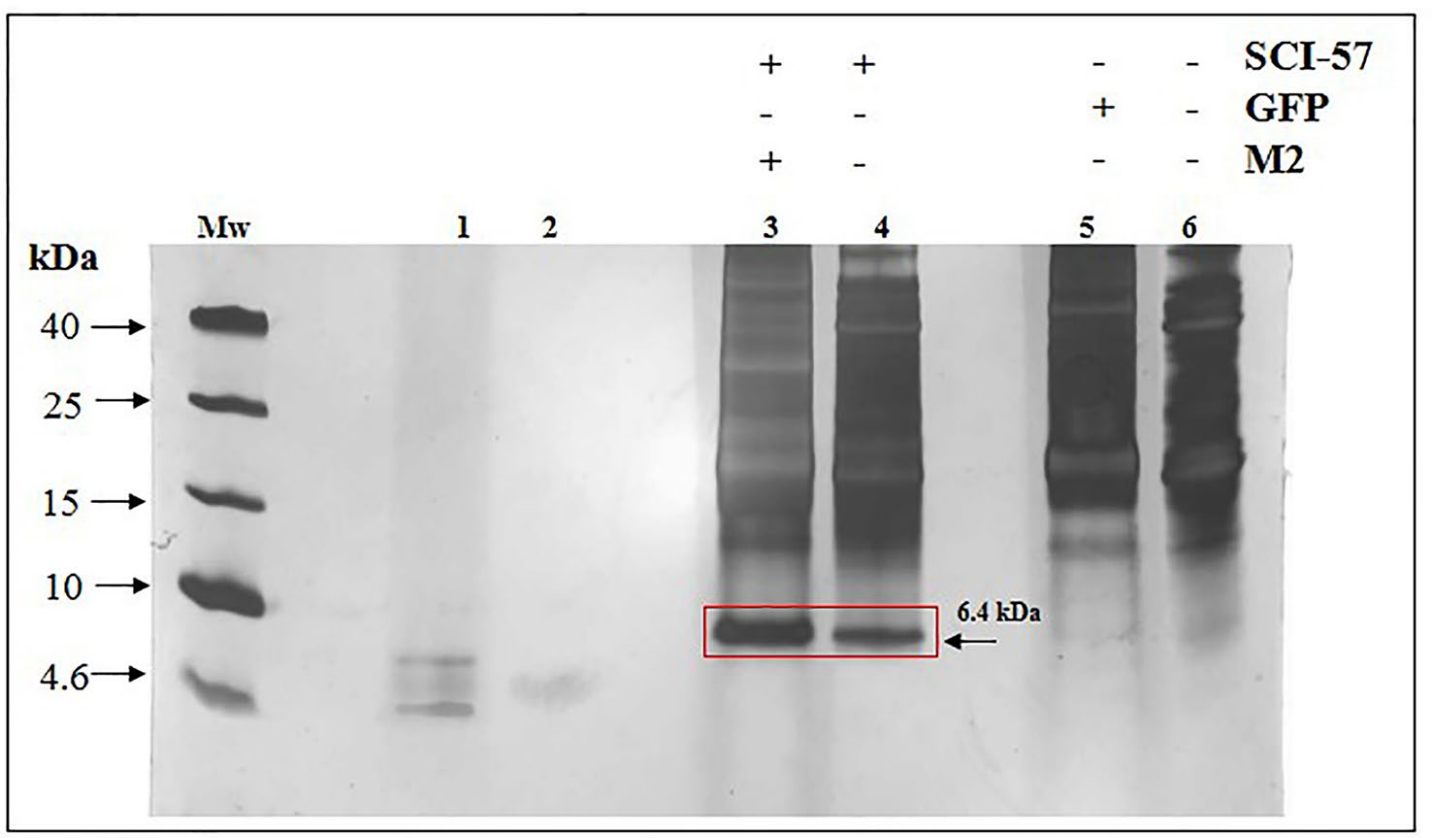
TSP analysis from NLEN and NLE from agroinfiltrated plants by SDS-PAGE. Twenty micrograms of TSP were loaded per lane. Mw, Molecular weight marker; lane 1, Insulin 0.01 µg; lane 2, Insulin 0.001µg; lane 3, NLE-SCI-57/M2; lane 4, NLE-SCI-57; lane 5, NLE-GFP; lane 6, NLEN. Side numbers indicate molecular mass markers in kDa. Red Square and black arrow indicates the protein corresponding to SCI-57.

To confirm the expression of SCI-57 in *Nb* plants, TSP extracts (TSPE) were separated by SDS-PAGE. A protein ∼6.4 kDa in size, corresponding to SCI-57 molecular weight, was observed. The band was identified on NLE-SCI-57 and NLE-SCI-57/M2. However, band intensity was greater when M2 was co-expressed (**Figure 2**). The band was not detectable in NLEN or NLE-GFP (**Figure 2**).

### Gene Analysis by Endpoint PCR and Quantitative Real-Time PCR (qRT-PCR)

**Figure 3:**
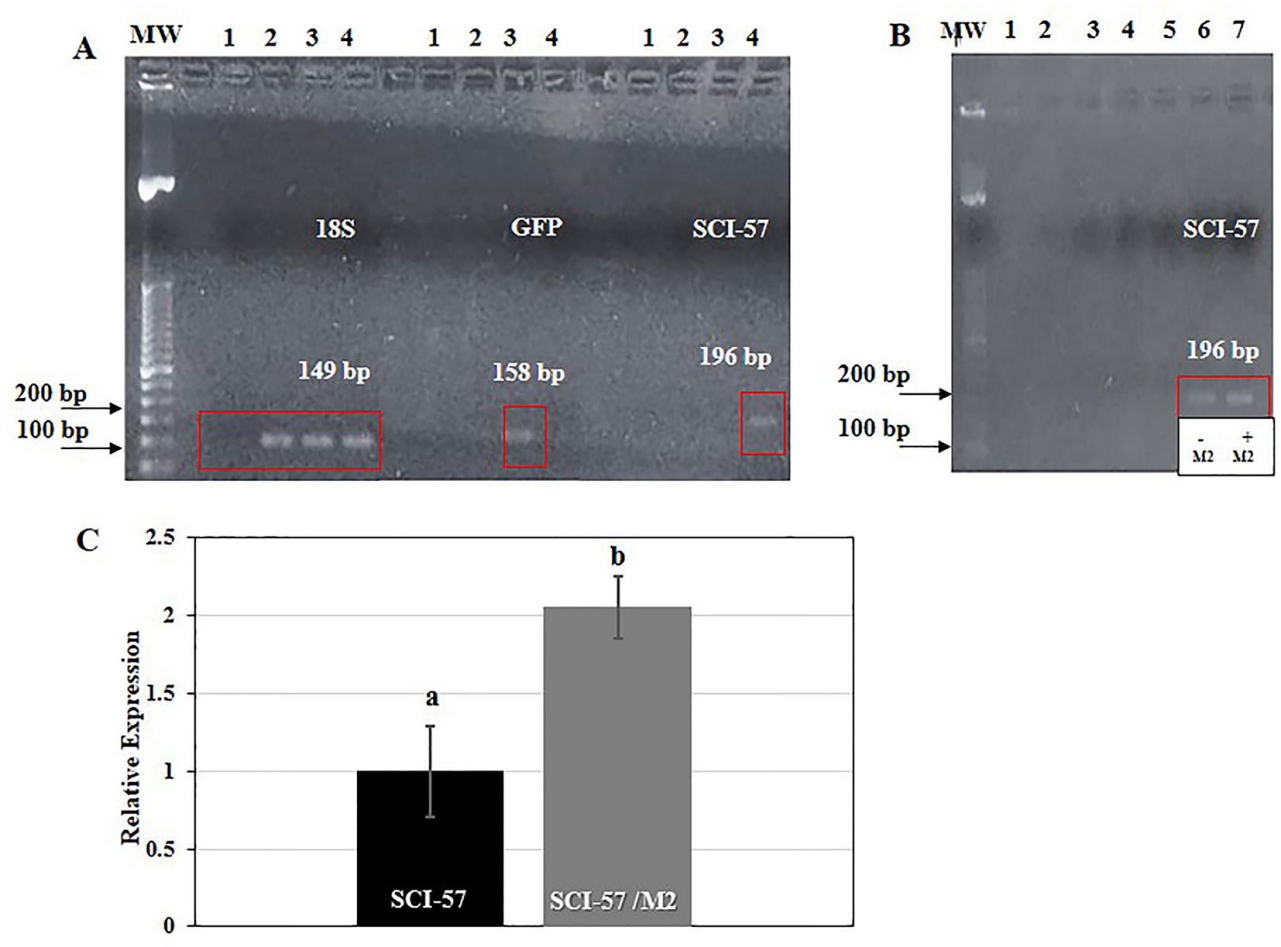
**(A)** RT-PCR analysis of 18S, GFP and SCI-57 gene expression. Reactions were performed in the same conditions for all lanes, with specific primer pairs: 18S (149 bp), GFP (158 bp) and SCI-57 (196 bp). Mw: Molecular weight marker; lane 1: No template controls (NTCs); lane 2: No-agroinfiltrated N.b; lane 3: Plants agroinfiltrated with GFP; lane 4: Plants agroinfiltrated with SCI-57. The red square indicates the location of the amplification products. **(B)** RT-PCR analysis of SCI-57 gene expression with and without M2 co-expression. Reactions were performed in the same conditions for all lanes, with specific primer pairs: SCI-57 (196 bp). Mw, Molecular weight marker; lane 3, NTCs; lane 4, No-agroinfiltrated N.b; lane 5, Plants agroinfiltrated with GFP; lane 6, Plants agroinfiltrated with SCI-57; lane 7, Plants agroinfiltrated with SCI-57 co-expressing M2. The red square indicates the location of the amplification products. **(C)** Analysis of the relative expression of SCI-57 with and without the M2 co-expression by qRT-PCR. Relative expression was evaluated employing the RT-PCR primers (**Supplementary Table S1**). Expression was normalized against 18S rRNA gene expression. Results are presented as the mean ± S.D. of three independent experiments in triplicate. Lowercase letters indicate significant differences according to the Mann-Whitney U test (p ≤ 0.05).

First, total RNA was extracted from the different *Nb* plants and reverse transcribed with random primers. The cDNA was then subjected to PCR with specific primer pairs: 18S rRNA (149 bp), GFP (158 bp) and SCI-57 (196 bp). The 18S rRNA was chosen as a housekeeping gene. The presence of 18S rRNA was confirmed in agroinfiltrated and non-agroinfiltrated plants, as expected. The presence of GFP was observed on lane 3 when GFP primers were used. This lane corresponds to *Nb* agroinfiltrated with pICH31070-GFP. Whereas SCI-57 was observed on lane 4 when SCI-57 primers were used, a lane which corresponds to *Nb* agroinfiltrated with pICH31070-SCI-57 (**Figure 3A**).

In **Figure 3B**, a slight difference in band intensity between SCI-57 expression in the presence and absence of M2 co-expression is apparent. Our observations indicate that M2 co-expression increases SCI-57 yield.

Relative expression quantitative real-time PCR was employed in order to quantify SCI-57, using cDNA from agroinfiltrated plants, both with and without co-expressed M2. Final relative quantification was carried out using the comparative 2^−ΔΔCT^ method. Expression levels were normalized using the 18S rRNA constitutive endogenous gene. The cDNA from agroinfiltrated plants with co-expressed M2 showed approximately two-fold change difference in SCI-57 level of expression compared with agroinfiltrated plants without co-expressed M2 (**Figure 3C**).

### SCI-57 Purification

**Figure 4:**
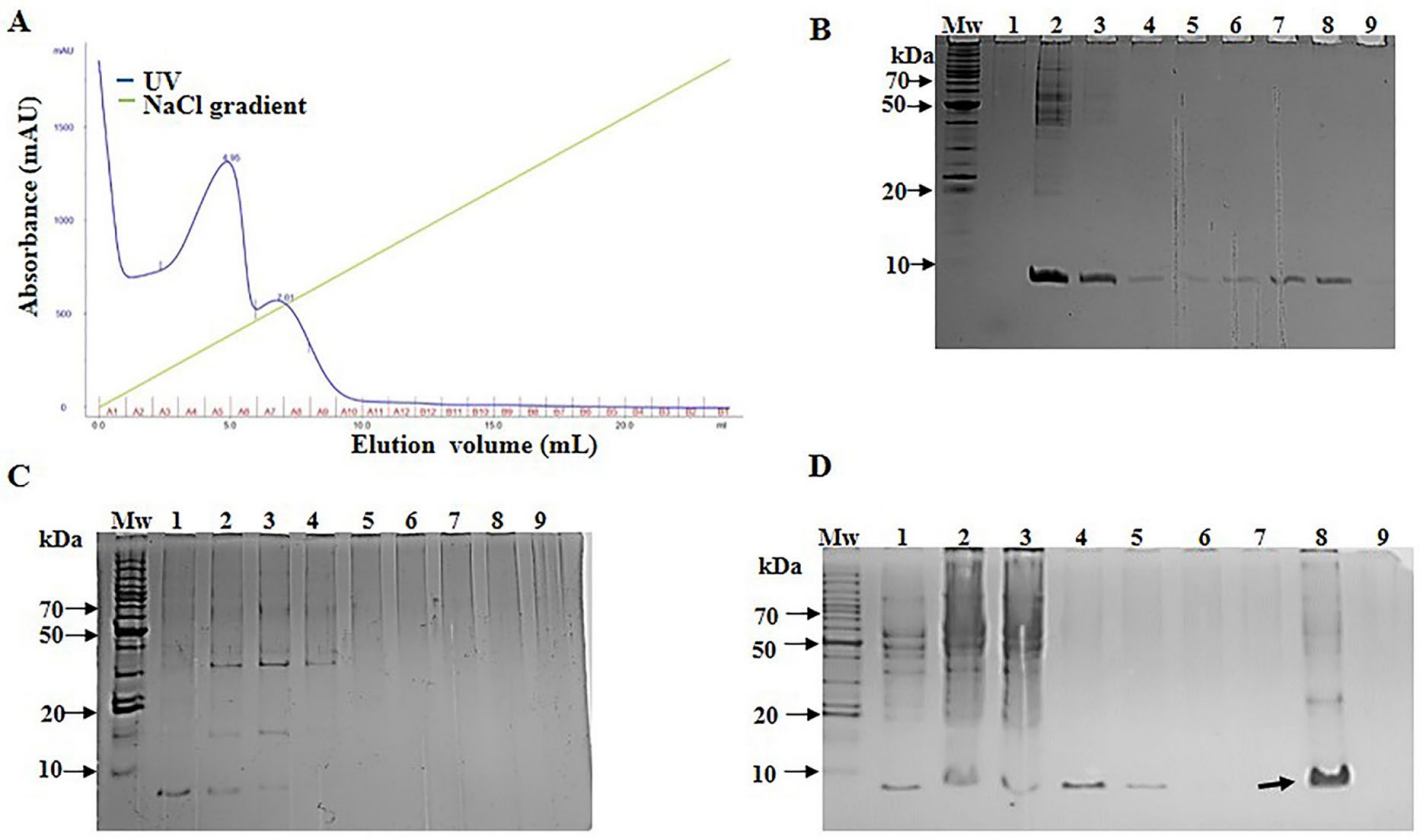
NLE-SCI-57 purification and fraction analysis. **(A)** NLE SCI-57 cation exchange separation performed on SP-Sepharose Fast Flow. Five ml of the sample was loaded. Elution fractions were 1 ml, and the flow rate was set at 0.4 ml/min 100 mM citrate buffer pH 4.3 (elution with 0–1 M linear NaCl gradient). Absorbance was expressed in milli-absorbance units for the A280 (blue line). Fractions were labeled by the A–B letters from 1–12 numbers. **(B)** 13% Coomassie stained Glycine SDS-PAGE gel, on which NLE-SCI-57 elution fractions were resolved. Mw: Molecular weight marker; lane 1:3.5 μg recombinant human insulin (PISA pharmaceutical, México); lane 2,3: Flow through from the column; from lane 4 to 8: Fraction A1 to A5 respectively from SCI-57 purification by cation exchange. **(C)** 13% Coomassie stained Glycine SDS-PAGE gel, on which NLE-SCI-57 elution fractions were resolved. Mw: Molecular weight marker; from lane 1 to 9: Fraction A6 to B11 respectively from SCI-57 purification by cation exchange. **(D)** 13% Coomassie stained Glycine SDS-PAGE gel, displaying ultrafiltrated A2–A7 fractions obtained by the SCI-57 purification by FPLC. Mw, Molecular weight marker, lane 1; NLE SCI-57, lane 2, NLE SCI-57 concentrated with 3 kDa membrane, lane 3: Flow through from the column, lane 4 to 7: Fraction A1 to A4, lane 8: Sample containing A2–A7 fractions ultrafiltrated with 3 kDa membrane, and analyzed by HPLC. 5 μL molecular weight marker and 20 μL of each fraction were loaded on the gel. Arrow indicates the protein corresponding to SCI-57.

**Figure 5:**
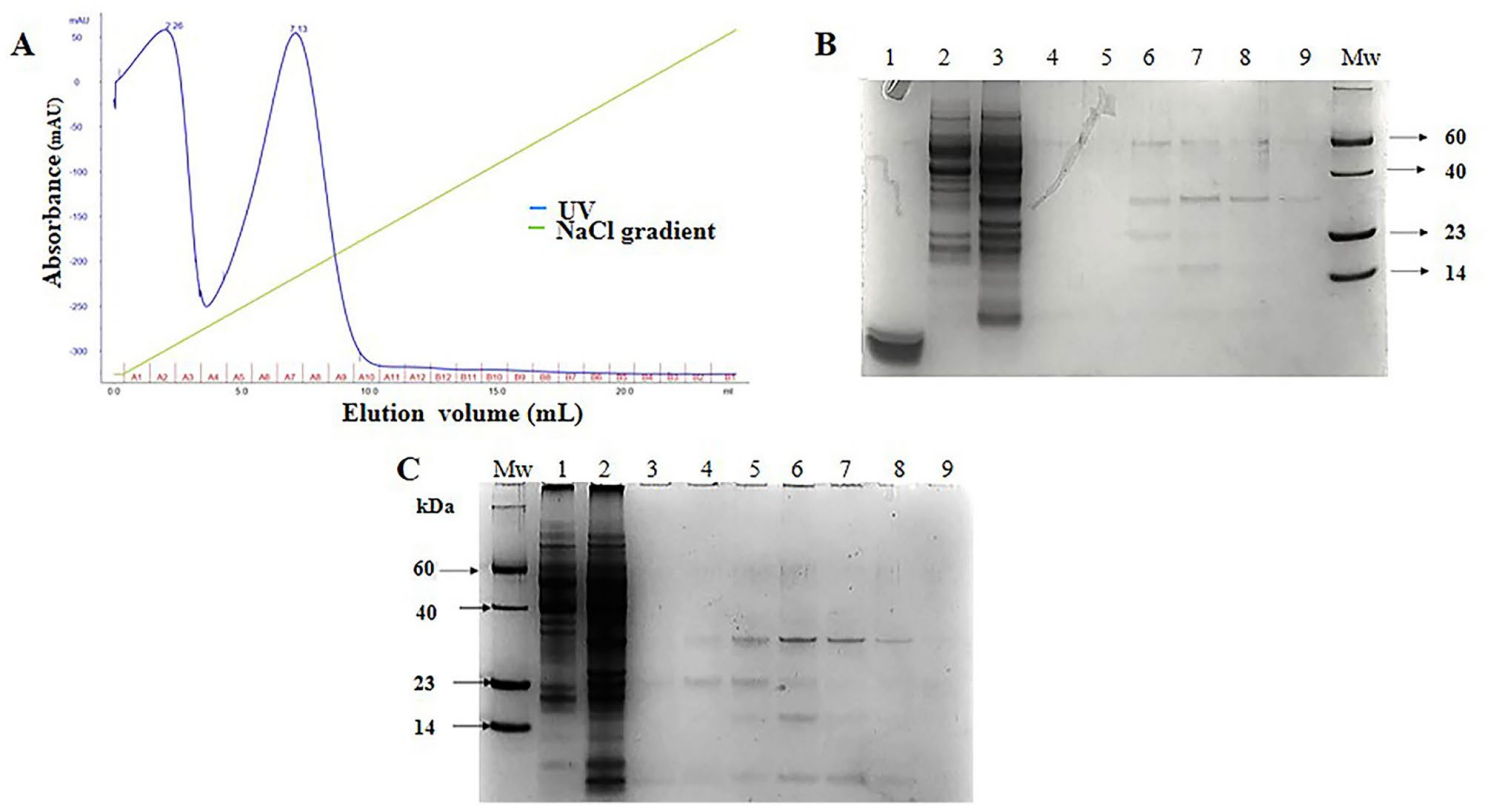
NLE-SCI-57/M2 purification and fraction analysis. **(A)** NLE-SCI-57/M2 cation exchange separation performed on SP-Sepharose Fast Flow 5 ml of the sample was loaded. Elution fractions were 1 ml, and the flow rate was set at 0.4 ml/min 100 mM citrate buffer pH 4.3 (elution with 0–1 M linear NaCl gradient). Absorbance was expressed in milli-absorbance units for the A280 (blue line). Fractions were labeled by the A–B letters from 1–12 numbers. **(B)** 13% Coomassie stained Glycine SDS-PAGE gel, on which SCI-57/M2 elution fractions were resolved. Lane 1, Insulin 10 µg, lane 2, NLEN, lane 3, NLE SCI-57/M2, lane 4, A1 fraction, lane 5, A2 fraction, from lane 6 to 9, -Fraction A5 to A8 respectively obtained by the purification with cation exchange FPLC, Mw, Molecular weight marker. **(C)** 13% Coomassie stained Glycine SDS-PAGE gel, on which SCI-57/M2 elution fractions were resolved. Mw, Molecular weight marker, lane 1, NLEN, lane 2, NLE SCI-57/M2, from lane 3 to 9, A3 to A9 fractions. 5 μL molecular weight marker and 20 μL of each fraction were loaded on the gel.

Purification of SCI-57 was carried out with a cation exchange column from the NLE-SCI-57 and NLE-SCI-57/M2 samples. The equipment allowed us to monitor the relative intensity at 280 nm of each fraction obtained (**Figures 4A** and **5A**).

High purity of SCI-57 is crucial when administered through direct injection into the blood of the diabetes patient. Correspondingly, SCI-57 purification was accomplished by cation exchange FPLC. The recombinant human insulin as a positive control was purified using ÄKTA avant 25 with prepacked SP-Sepharose Fast Flow. Insulin was eluted, displaying one peak on the chromatogram, from the fraction A4 to fraction A11 (**Supplementary Figure S2**). The aforementioned fractions corresponded to 0.17 M to 0.42 M NaCl. This is consistent with previous publications which establish that, under similar experimental conditions, insulin captured by the column was eluded from 0.10 to 0.45 M NaCl, recovering up to 70% (Jagschies et al., 2018). It was thus probable that SCI-57 would be eluted with a similar concentration of NaCl.

With respect to NLE-SCI-57 purification, two peaks were observed: one from A2–A6 and another from A7–A10, both eluting at the same NaCl concentration range as the insulin peak (**Figure 4A**). SDS-PAGE analysis from fraction A2–A5 (**Figure 4B**) displays one band corresponding to the insulin analog molecular weight ∼ 6.4 kDa. SDS-PAGE analysis from the fractions conforming to the second peak (A6–A10) show a band corresponding to SCI-57 molecular weight, as well as several bands that may represent higher molecular weight proteins (**Figure 4C**). The SCI-57 recovery was greater than 60% of the protein captured by the column (**Table 2**). Regarding NLE-SCI-57/M2 purification, two peaks were observed in the chromatograms (**Figure 5A**). The first peak goes from fraction A1–A3 and the second peak from A4–A10. The A1–A10 fraction analysis (**Figures 5B, C**) detected a band corresponding to a protein with the size ∼6.4 kDa and the presence of proteins with a molecular weight greater than 14 kDa, which was similar to the proteins observed on A7–A10 NLE-SCI-57 purification. The SCI-57/M2 recovery was 46% of the protein captured by the column (**Table 2**).

After identifying the fractions containing SCI-57, the elution fractions in which the protein is present were combined, and the resultant solution was concentrated twenty-fold in a 3-kDa cutoff filter (Millipore, Bedford, MA, USA). The resultant solution contains pure SCI-57, as revealed by the gel (**Figure 4D**, lane 8).

### Reversed-Phase High-Performance Liquid Chromatography (RP-HPLC) Analysis

**Figure 6:**
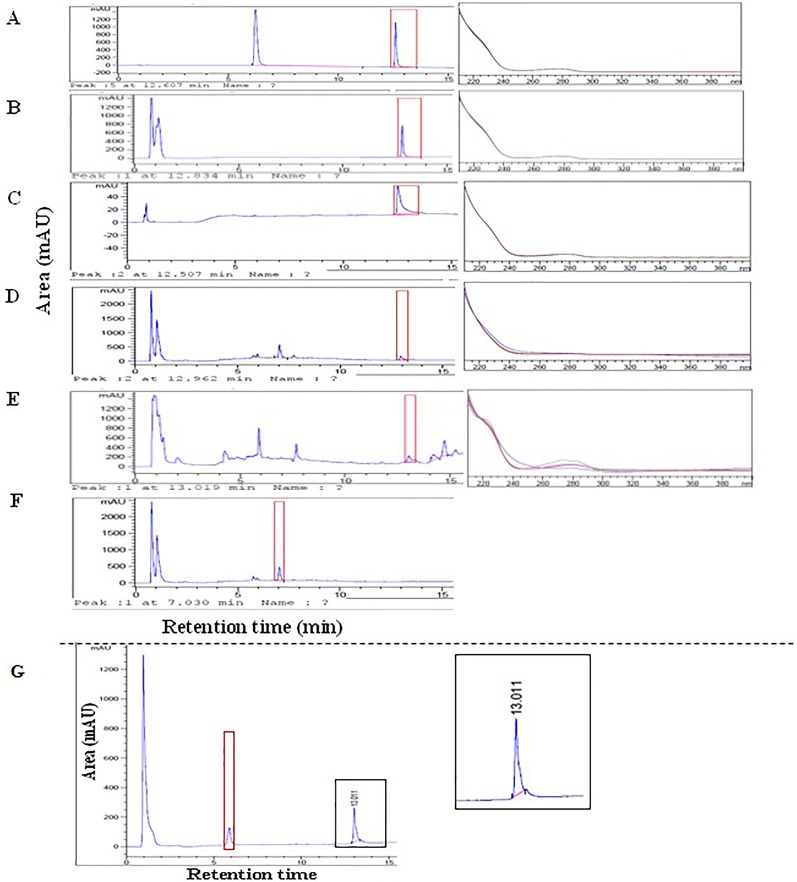
Chromatograms and UV spectra respectively from: **(A)** Commercial insulin 0.12 mg/ml (PISA, pharmaceutics); **(B)** EP insulin 0.2 mg/ml; **(C)** Bovine insulin 0.125 mg/ml; **(D)** NLE SCI-57; **(E)** NLE SCI-57 M2; **(F)** NLEN; **(G)** Chromatogram from NLE-SCI-57 (A2–A7 fractions) purification and concentration by ultrafiltration with 3 kDa membranes. All samples were diluted on citrate buffer pH 4.3.

Once SCI-57 has been identified in the NLE-SCI-57, samples are evaluated by RP-HPLC. As a positive control, EP insulin, commercial insulin and bovine insulin were used. NLE-SCI-57 (**Figure 6D**) and NLE-SCI-57/M2 (**Figure 6E**) samples exhibit a peak with a retention time (RT) = 12.96 ± 1.30 and RT = 13.02 ± 1.30 min, which is similar to commercial insulin RT = 12.61 ± 1.26 (**Figure 6A**), EP insulin RT = 12.83± 1.28 (**Figure 6B**), and bovine insulin RT = 12.51± 1.25 (**Figure 6C**). The peak was not detectable in extracts of non-agroinfiltrated plants (**Figure 6F**).

After evaluating NLE-SCI-57, a purified SCI-57 sample from A2–A7 fractions was analyzed with the help of HPLC (**Figure 4D**, lane 8) to confirm that the peak observed on NLE-SCI-57 corresponds to the insulin analog SCI-57. The chromatogram (**Figure 6G**) displays a peak with a rt = 13.011, corresponding to the SCI-57 retention time previously observed on non-purified NLE-SCI-57.

### Ionization Pattern and Exact Molecular Weight Determination

**Figure 7:**
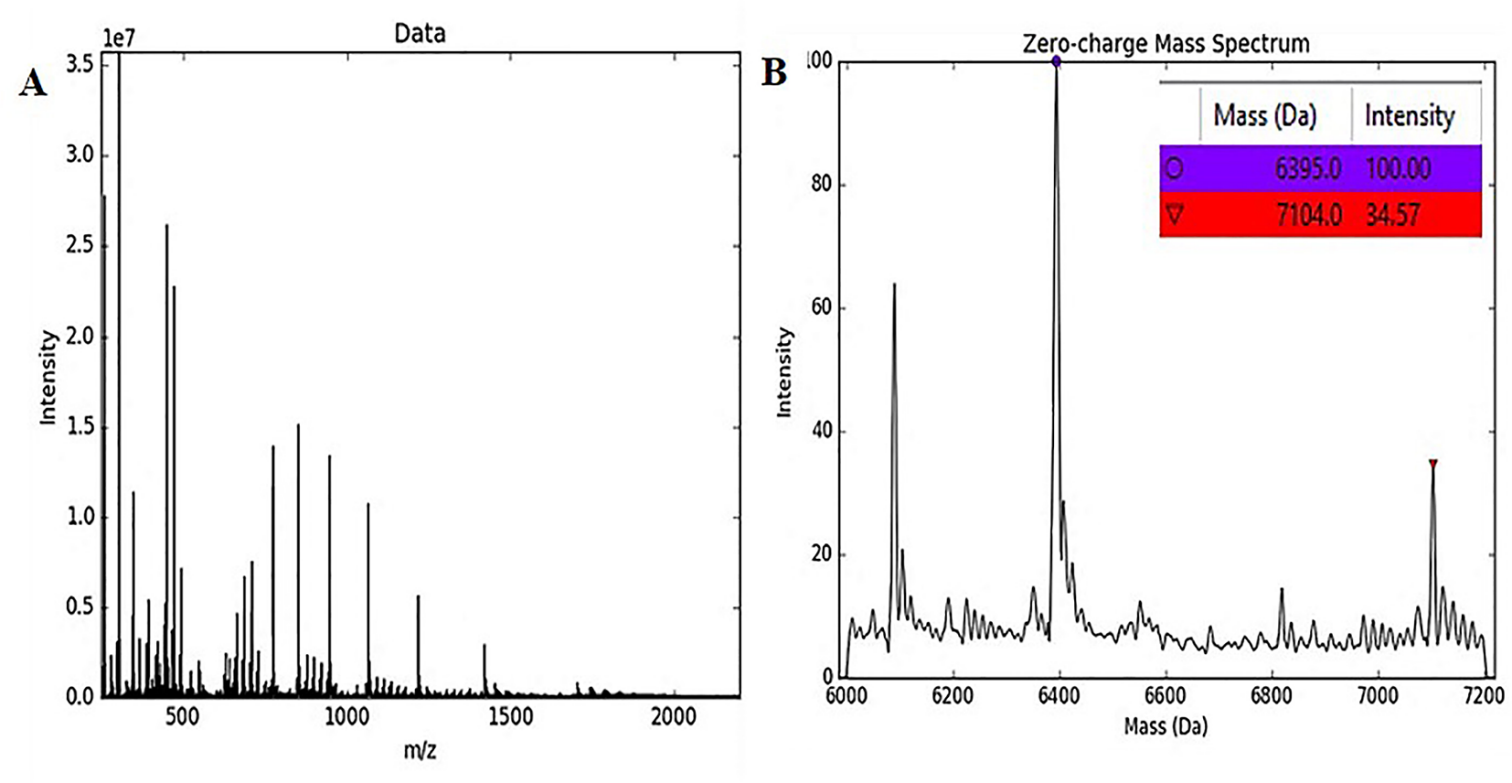
MS spectra and molecular weight of native protein. **(A)** The spectrum obtained by MS of the SCI-57 purified fractions (A2–A7) containing SCI-57 in its native form. **(B)** Molecular weight of proteins contained in SCI-57 purified fractions, by applying the deconvolution to the spectrum obtained by MS.

An SCI-57 purified protein sample (A2–A7 fractions) was analyzed using a SYNAPT-HDMS system (Waters Corp.) with ESI-Lockspray interphase. Molecular weight estimations were generated by the UniDec GUI tool version 1.1.10 (Marty et al., 2015). Two SCI-57 proteins variants were identified from the sample (**Figure 7**). The protein variant with highest intensity had a molecular weight corresponding to SCI-57 (∼6.4 kDa). The second protein variant had a molecular weight of ∼ 7.1 kDa. The difference of mass units between the SCI-57 insulin analog and the protein mass identified is likely to refer to the addition of KDEL peptide to the protein sequence. The EP insulin experimental molecular weight was ∼5.8 kDa (**Supplementary Figure S4**).

**Figure 8:**
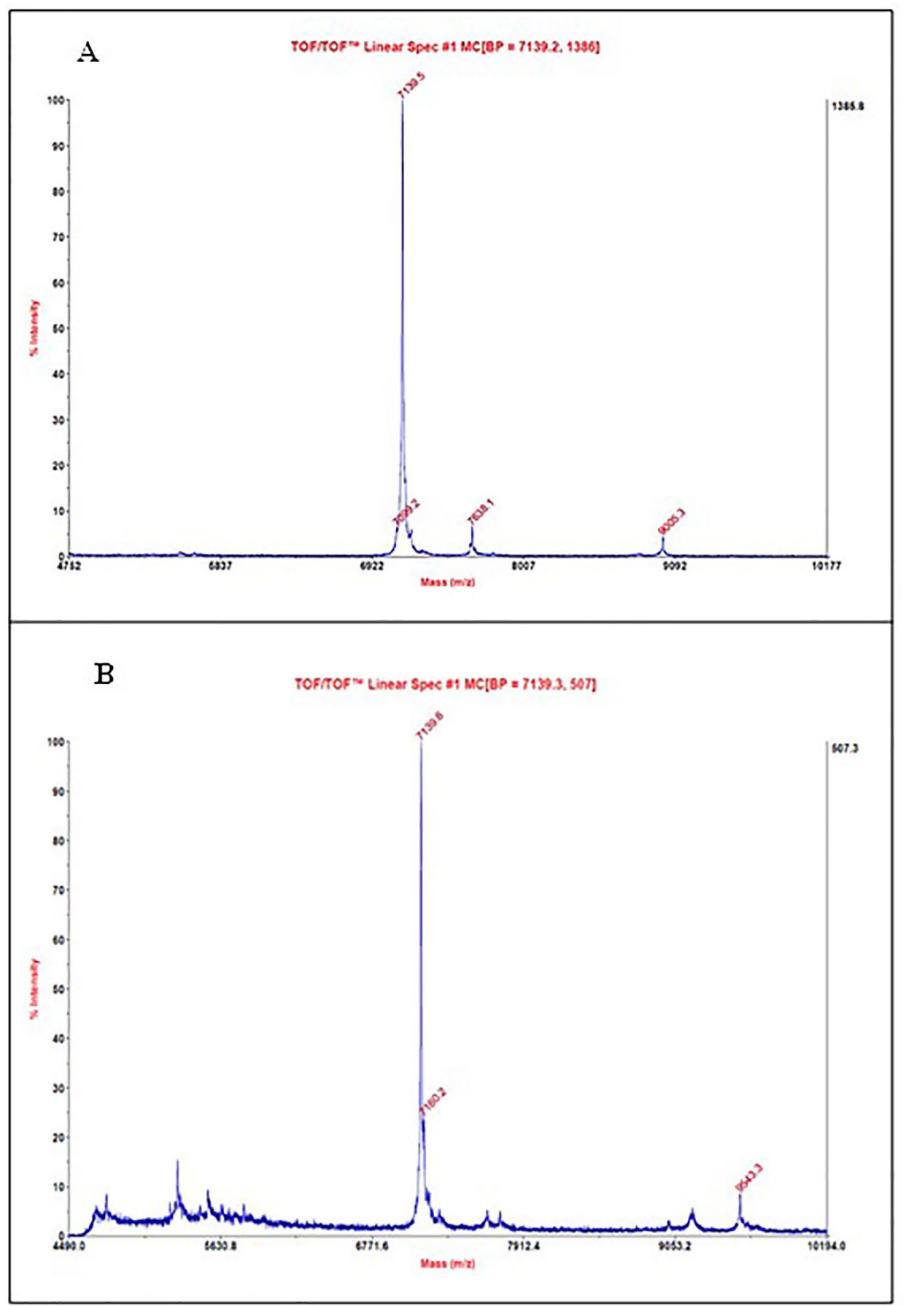
MALDI-TOF mass spectrum from **(A)** A2–A4 fractions obtained by FPLC of NLE SCI-57/M2. **(B)** A5–A7 fractions obtained by FPLC of NLE SCI-57/M2. Molecular masses were determined by MS using vendor-provided software (Applied Biosystems/MDS SCIEX, USA).

The samples from fractions A2–A4 and A5–A7 NLE-SCI-57/M2 and EP insulin were analyzed using a MALDI TOF/TOF Analyzer 4800 plus mass spectrophotometer (Applied Biosystems/MDS SCIEX, USA), using a linear mode with a laser intensity of 3,800 Hz. On the A2–A4 purified fractions, the presence of three proteins was identified; the most abundant on the sample had a molecular weight of ∼7.1 kDa (**Figure 8A**). The protein was previously identified on the purified protein sample from NLE-SCI-57, probably representing the one corresponding to a molecular weight of SCI-57 + KDEL peptide. On the A5–A7 purified sample, the ∼7.1 kDa protein was also identified (**Figure 8B**). The EP insulin molecular weight was ∼5.8 kDa (**Supplementary Figure S5**).

### Identification of SCI-57 and Proteomic Profile by Gel-Assisted Sample Preparation (GASP), Using Liquid Chromatography-Tandem Mass Spectrometry (LC-MS/MS) and Applying Sequential Windowed Acquisition of All Theoretical Fragment Ion Mass Spectra (SWATH)

We performed a differential proteomic NLE analysis on plants expressing heterologous proteins (GFP, SCI-57, and SCI-57/M2) and NLEN to identify the proteomic changes related to heterologous protein expression on the TSP extract. A total of 280 plant proteins were detected with at least two unique peptides. The 280 protein intensities on NLEN sample were used to normalize protein intensities on NLE of plants expressing heterologous proteins in order to determine relative protein expression.

On a TSP basis, 102 proteins were up-regulated, and 82 down-regulated in NLE-GFP, compared to non-agroinfiltrated leaves; similar to NLE-SCI-57, with 89 up-regulated proteins and 102 down-regulated. NLE-SCI-57/M2 manifested 103 up-regulated and 44 down-regulated proteins. The plants expressing heterologous proteins (GFP, SCI-57 and SCI-57/M2) share 48 up-regulated and 23 down-regulated proteins by at least twofold (**Supplementary Tables S2**, **S3**), work is also underway to address these proteins to the most affected biological processes in leaves. Interestingly, all the plants expressing heterologous proteins showed up to five-fold up regulated pathogenesis-related and stress-inducible proteins (**Table 1**) which are related to agroinfiltration as previously reported (Pruss et al., 2008; Goulet et al., 2010; Robert et al., 2015); for example: protease inhibitors (Kunitz 2 trypsin inhibitor), cell wall modifying enzymes (Expansin) and proteins related to the pathogenesis (PR-) induced by *Agrobacterium* mainly PR-1 proteins (antimicrobial activity), PR-2 (β-glucanases) and PR-3 (chitinases).

**Table 1:** Relative abundance of up-regulated proteins^1^ by at least 5-fold in NLE GFP, NLE SCI-57 and NLE SCI-57/M2 compared with NLEN.

NCBI accession	Protein name	Relative abundance (n-fold)		
		$\frac{\mathrm{NLE}\;\mathrm{GFP}}{\mathrm{NLEN}}$	$\frac{\mathrm{NLE}\;\mathrm{SCI}-57}{\mathrm{NLEN}}$	$\frac{\mathrm{NLE}\;\mathrm{SCI}-57/M2}{\mathrm{NLEN}}$
XP_009760305.1	Glucan endo-1,3-beta-glucosidase, basic vacuolar isoform isoform X2	56.6	15.4	19.7
XP_009617302.1	Endochitinase A	69.5	37.6	36.2
XP_019225618.1	Pathogenesis-related R major form	6.8	6.3	7.1
XP_009781670.1	Pathogenesis-related protein R minor form	15.6	18.8	15.5
XP_016495116.1	Suberization-associated anionic peroxidase-like	58.7	8.8	64.5
XP_019244706.1	Kunitz trypsin inhibitor 2-like	72.4	50.5	53.1
XP_009593782.1	Peroxidase P7-like	31.1	8.7	24.0
AAA34078.1	Beta(1,3)-glucanase regulator	32.5	34.5	37.3
CBK52316.1	Nb cell deth marker	9.3	13.3	15.2
XP_019238089.1	Basic form of pathogenesis-related protein 1	27.7	5.8	43.6
XP_019261786.1	Wound-induced protein WIN1	31.7	7.4	49.1
XP_009768114.1	Expansin-like A2	7.7	13.8	9.1
OIT28720.1	Basic endochitinase	23.2	10.6	6.7
XP_019265536.1	Non-specific lipid transfer protein GPI-anchored 1	15.1	15.5	9.7
XP_019241188.1	Aquaporin PIP-type pTOM75	13.3	20.3	6.6
XP_009787159.1	Basic form of pathogenesis-related protein 1-like	313.3	81.0	128.3
prf||1202235A	protein 1a,pathogenesis related	22.1	35.2	10.9

^1^Proteins were listed base on the n-fold expression normalized with NLEN. Ratios were inferred from MS/MS peptide abundance values determined for each protein. Red numbers indicate the group with the highest value and blue values indicate the group with the lowest value.

Label-free analysis made it possible to identify the peptide FVNQHLCGSDLVEALYLVCERG on samples from plants expressing SCI-57 by measuring the intensity of the seven most intensive peptide fragment ions of the peptide. This peptide is also one of the identified peptides for the peptide map of insulin digestion with trypsin. The peptide was not detected on NLEN (**Supplementary Figure S6**).

### Diagonal Electrophoresis for Detecting Disulfide Bridges

**Figure 9:**
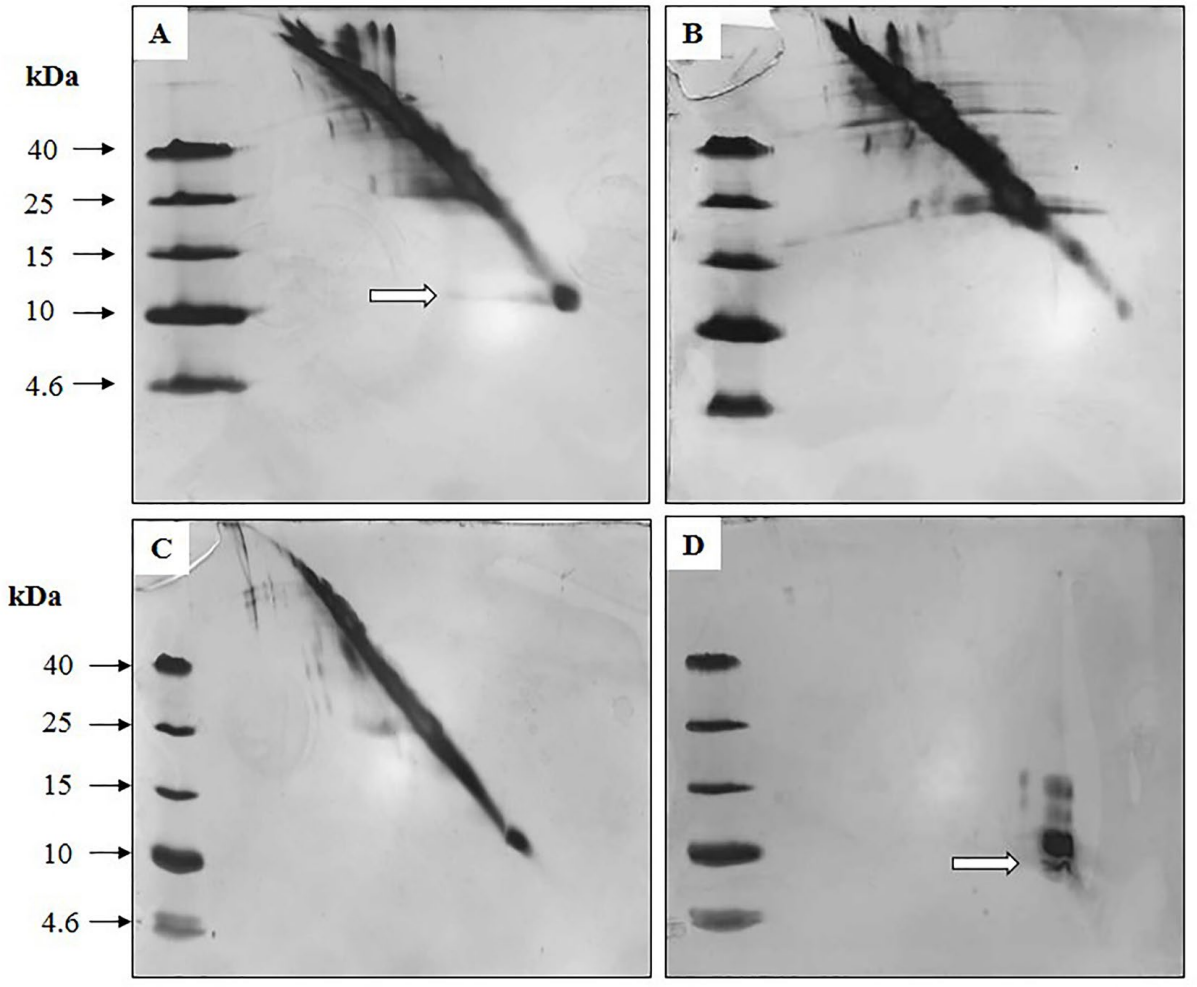
Silver-stained diagonal gel, identifying intermolecular protein disulfides in **(A)** NLE SCI-57, **(B)** NLEN, **(C)** NLE SCI-57/M2 and **(D)** EP insulin. Eight microgram of the samples and 1 µg of insulin were loaded on the gel. White arrows indicate proteins with disulfide bridges.

**Figure 9** exhibited diagonal electrophoresis gels stained with silver, evaluating the presence of disulfide bridges in the NLE-SCI-57, NLE-SCI-57/M2, NLEN, and insulin samples. Spots or bands that ran off the diagonal and to the left of the slope indicated proteins containing disulfide bridges. Therefore, NLE-SCI-57 (**Figure 9A**) showed a protein corresponding to SCI-57 molecular weight, running off the diagonal to the left of the slope and indicating the presence of disulfide bridges. The NLE-SCI-57/M2 displayed a protein corresponding to SCI-57 molecular weight, however, it did not run off the diagonal (**Figure 9C**).

### SCI-57 Detection by Anti-Insulin Antibodies

Quantitative determination of SCI-57 was accomplished by applying the enzyme-linked immunosorbent assay (ELISA) method. The response was measured using the commercially available insulin ELISA kit (DRG Insulin ELISA EIA-2935). The SCI-57 concentration was obtained by equation y = 38.867x − 26.925. The equation was obtained by plotting the absorbance values against insulin concentrations from the calibrator solutions provided in the kit, with a correlation coefficient of 0.9967. Purified SCI-57 obtained from NLE-SCI-57 A2–A7 fractions manifested a concentration of 1.16^−6^ mg/ml (26.65 mU/L). The sample was measured using the Bradford method with a concentration of 0.086 mg/ml. This concentration corresponds to 19.11% of total soluble protein (%TSP) or 0.1505 mg/g leaves fresh weight (LFW) (**Table 2**).

**Table 2:** Yield of the SCI-57 expression and purification.

Protein	Recovery from the purification method (%)	Highest expression level	Plants/g recombinant protein
SCI-57	62.52	19.11% TSP or 0.1505 mg/g LFW	2,293^1^
SCI-57/M2	46.39	ND	ND

The expression levels are reported as mass of the insulin analog SCI-57 per unit of biomass (LFW, leaves fresh weight.). The recombinant protein productivity values were calculated by considering leaf biomass yield per plant [^1^ (Lai and Chen, 2012)]. ND, not determined.

### SCI-57 Affects 3T3-L1 Adipogenesis

**Figure 10:**
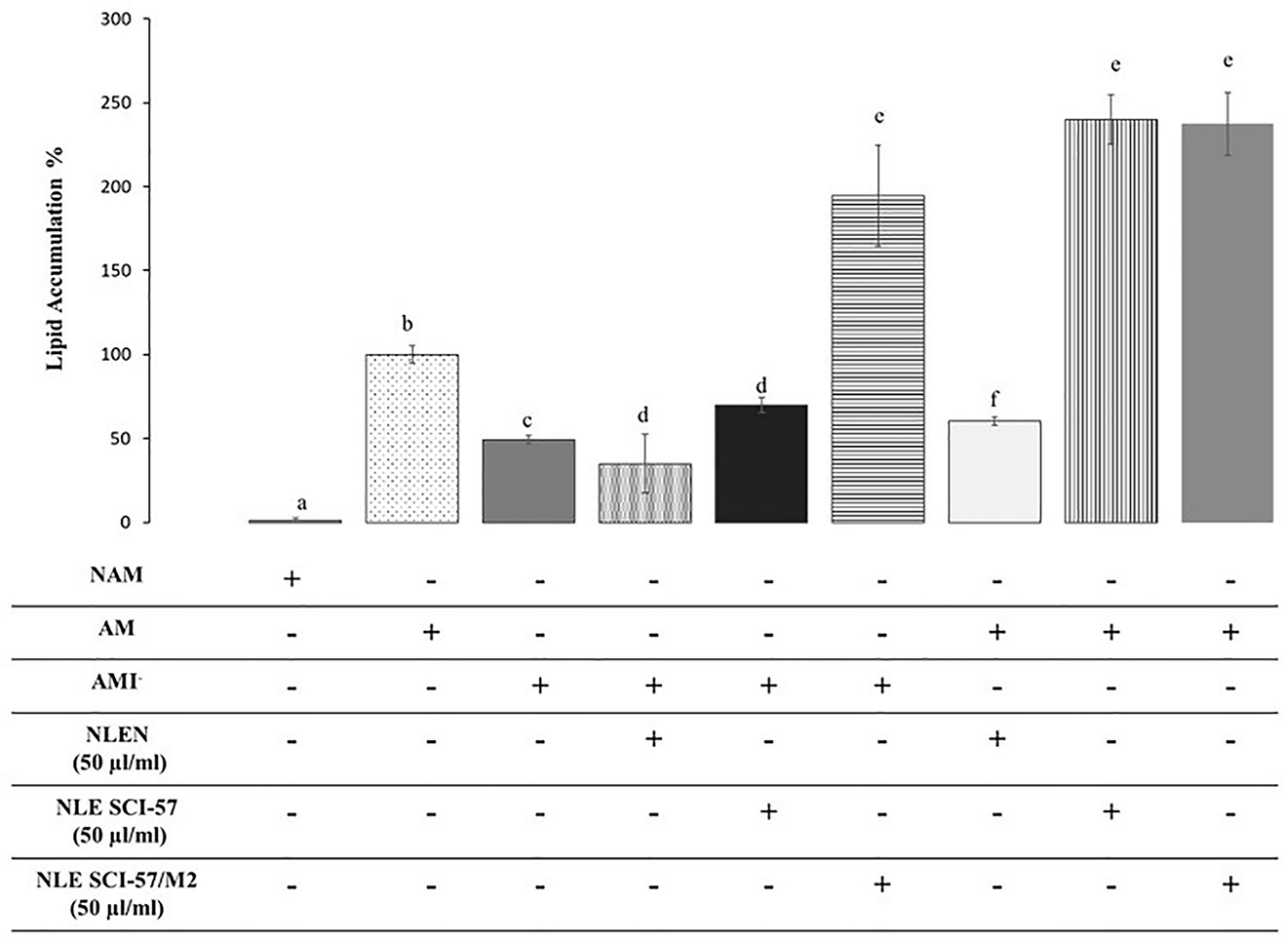
Effect of SCI-57 on 3T3 adipogenesis. Adipose differentiation of 3T3-L1 preadipocytes was induced in the presence (AM) or the absence (AMI^−^) of 5 μg/ml insulin. Control cultures received a non-adipogenic medium (NAM). Effects of NLE preparations on 3T3-L1 adipogenesis were evaluated under both induction conditions by quantitation of intracytoplasmic lipid accumulation with oil red O. Results are presented as the mean ± S.D. of experiments in triplicate. Lowercase letters indicate significant differences according to the Mann-Whitney U test (*p* ≤ 0.05).

In order to determine whether *Nb* preparations affect the development of adipose tissue, we evaluated their effects on 3T3 adipogenesis. When added to preadipocytes exposed to insulin-lacking adipogenic medium (AMI^−^), NLE-SCI-57 stimulated the accumulation of lipids in these cells significantly compared to the insulin-lacking control (AMI^−^), although to a lesser extent than the positive control adipogenic medium (AM). This proadipogenic effect was stronger in NLE-SCI-57/M2 treated cells and was not observed in cells treated with (NLEN) (**Figure 10**).

We then assayed the effects of NLE-SCI-57 and NLE-SCI-57/M2 in insulin-containing medium or adipogenic medium (AM) to determine whether the extracts have synergic effect on adipogenesis stimulation (**Figure 10**). Under these conditions, NLE-SCI-57 and NLE-SCI-57/M2 increased the pro-adipogenic effect of insulin. As in the previous assay, NLEN did not show proadipogenic effects.

### SCI-57 Stimulates 2-NBDG Uptake

**Figure 11:**
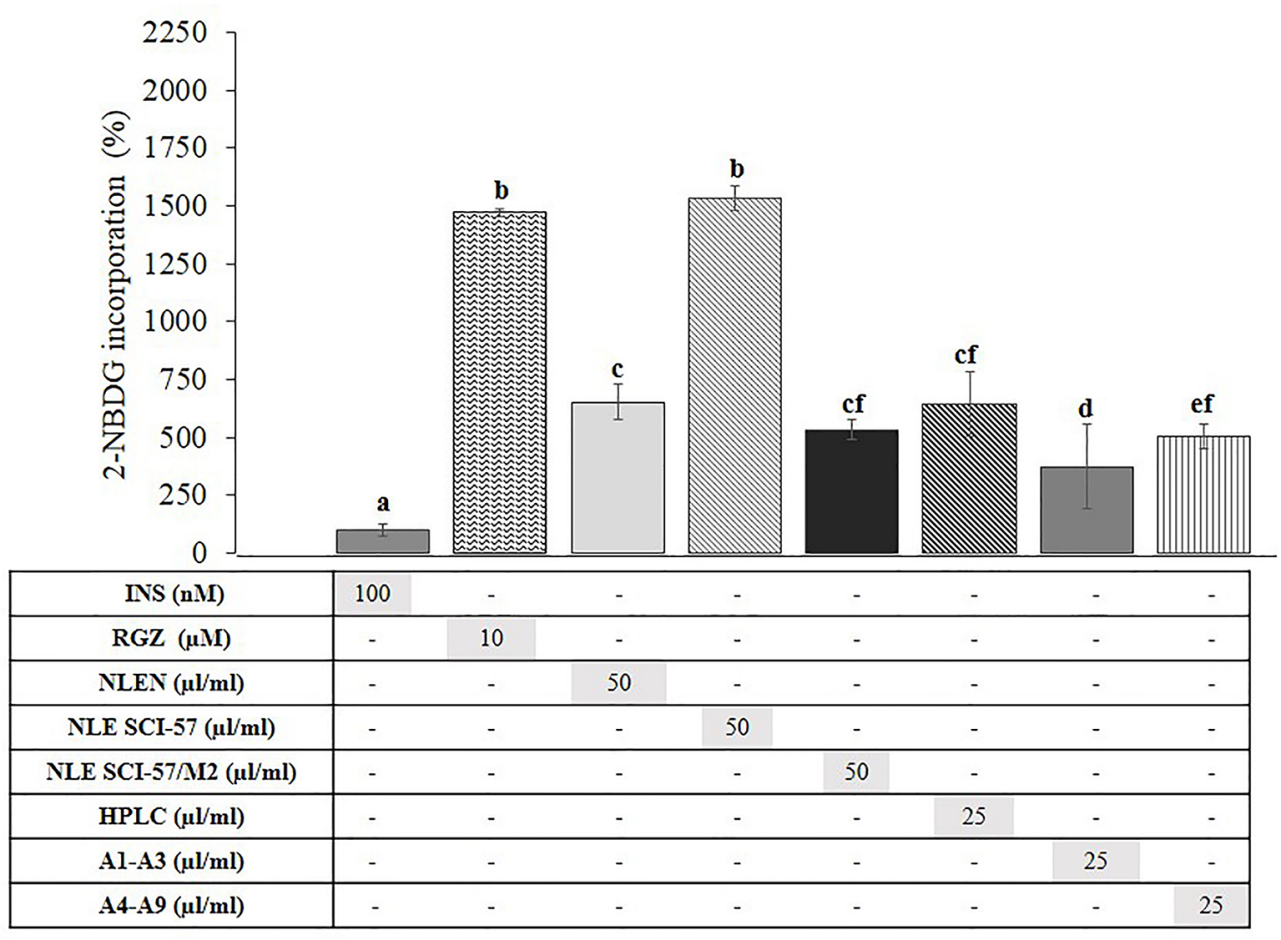
Effect of NLEN, NLE SCI-57, NLE SCI-57/M2 and purified samples on 2-NBDG uptake in 3T3-L1 adipocytes. Mature 3T3-L1 adipocytes were incubated as described (Zapata-Bustos et al., 2014b). Control treatments were incubated with insulin 100 nM (INS) or rosiglitazone 10µM (RGZ). Results are presented as the mean ± S.D. of experiments in quadruplicate. Lowercase letters indicate significant differences according to the Mann-Whitney U test (*p* ≤ 0.05). HPLC: Purified NLE-SCI-57 sample (A2–A7 fractions) analyzed by HPLC (**Figure 6G**). A1–A3 and A4–A9: NLE-SCI-57/M2 fractions.

We evaluated the effect of SCI-57 on 2-NBDG incorporation in terminally differentiated 3T3-L1 cells to establish whether it stimulates glucose uptake by adipocytes (**Figure 11**). NLE-SCI-57 exerted a marked ∼15.3-*fold* increase in the 2-*NBDG* uptake in comparison to insulin, whereas NLE-SCI-57/M2 exhibited a 5.3-fold increase. The NLEN stimulated 2-NBDG incorporation, causing a 6.5-fold increase.

A purified NLE-SCI-57 sample (A2–A7 fractions) stimulated 2-NBDG incorporation by a 6.4-fold increase; the sample was previously analyzed by HPLC (**Figure 6G**). The purified fraction A1–A3 from NLE-SCI-57/M2 had a lower 2-NBDG uptake (3.7-fold increase) compared to the A4–A9 fraction (5.1-fold increase).

## Discussion

In this work, we have demonstrated that it is feasible to produce biologically active insulin analog SCI-57 by transient expression in *N. benthamiana* with a yield of 19.11% of TSP (**Table 2**). Furthermore, the number of plants for the production of one gram of the recombinant protein was calculated by considering leaf biomass yield per plant (Lai and Chen, 2012). Then, it will be necessary at least 2293 plants to get a gram of the insulin analog SCI-57, which is comparable to previous reports where the best-performing plant-based platforms (Merlin et al., 2014).

A ∼6.4 kDa band, which was expected to be the size of the insulin analog SCI-57, was detectable in the *N. benthamiana* leaf extract (NLE) from agro-infiltrated plants with SCI-57 constructions (**Figure 2**). Unpurified SCI-57 was readily detectable by SDS-PAGE according to Virgen-Ortiz and colleagues, although only abundant proteins are detected when using this technique (Virgen-Ortiz et al., 2013). Therefore, SCI-57 was expressed at significant levels. Furthermore, M2 co-expression tends to increase SCI-57 levels on leaf extracts. This was corroborated by gene analysis, as twice as much SCI-57 seemed to be expressed when M2 is co-expressed (**Figure 3C**).

The endoplasmic reticulum (ER) of plant cells is able to accumulate a high concentration of proteins with the addition of a retention signal (KDEL) at the carboxyl terminal (C-terminal) end of the recombinant protein; this retention signal was included in the SCI-57 gene sequence. However, Okomoto et al. (Okomoto et al., 1999) found that, on the second day of infection, the recombinant protein still possessed the KDEL retention sequence. From the third day onwards, a protein mixture without KDEL and with KDEL was present. On day 4, the KDEL sequence was removed from the recombinant protein.

Based on Okomoto’s findings and the MS analysis of the sample containing A2–A7 fractions from NLE-SCI-57 purification (**Figure 7**), we presume that the addition of a retention signal (KDEL) generates a mixture of two proteins: SCI-57 and SCI-57+KDEL. This concurs with the findings on the FPLC, where two peaks were observed (**Figure 4A**). Therefore, the band observed on the SDS-PAGE analysis (**Figure 4B**) from the fractions conforming to the first peak (A2-A6) corresponds to SCI-57 ∼6.4 kDa, whereas the bands with higher molecular weight observed on A7-A10 may be SCI-57+KDEL ∼7.1 kDa, which tends to form molecular aggregates (**Figure 4C**). Thus, SDS-PAGE was not capable of separating proteins with similar weights: SCI-57 and SCI-57+KDEL. Diagonal electrophoresis results (**Figure 9**) indicate that SCI-57 structure had disulfide bonds; hence, transient expression did not affect proper SCI-57 folding or disulfide bond formation.

M2 co-expression was used to enhance the yield and quality of SCI-57. However, the results from molecular weight determination (**Figure 8**) and diagonal electrophoresis suggested the presence of a 7.1 kDa protein with no disulfide bridges (**Figure 9**). Previous publications reported that M2 co-expression increases the pH in the Golgi apparatus (Jutras et al., 2015). Although SCI-57 mRNA was higher when M2 was co-expressed, it was believed that the use of M2 interferes in the binding of the KDEL protein to its receptor, neutralizing the acid pH necessary to carry out the binding. Basic pH inhibits the cleavage of the KDEL peptide (Reithmeier, 1996; Lodish et al., 2008) in the SCI-57 sequence. Thus, when M2 is co-expressed, SCI-57+KDEL production is enhanced, when compared to SCI-57 with no M2 co-expression. Therefore, the two peaks on the FPLC chromatogram may correspond to different degrees of SCI-57+KDEL aggregation. It is likely that on the A1–A4 fractions (first peak on the chromatogram, **Figure 5A**), the monomeric SCI-57+KDEL form was predominant; whereas on the A4–A9 fractions (second peak on the chromatogram, **Figure 5A**), oligomeric SCI-57+KDEL forms can be observed on the SDS-PAGE. Insulin self-assembly and aggregation were induced by our working conditions for purification (**Supplementary Figure S3**); previous publications reported that buffer citrate, chloride ions from NaCl, and the presence of zinc promote insulin aggregation (Carpenter, 1966; Nettleton et al., 2000). High NaCl concentration and zinc presence on the NLE-SCI-57/M2 could stimulate greater oligomeric formation on SCI-57+KDEL; thereby, interfering with the insulin analog SCI-57 recovery on the purification method.

From RP-HPLC, it was established that the band detected on SDS-PAGE gel from NLE-SCI-57 corresponded to a protein with similar retention time and UV spectra as the insulins that we evaluated (**Figure 6**). These findings were corroborated by the analysis of purified A2–A7 fractions (**Figure 6G**). In the case of the NLE-SCI-57/M2 sample, the presence of a peak with a similar retention time is also observed (**Figure 6E**). However, the UV spectrum differs slightly from the NLE-SCI-57 sample. Therefore, M2 protein interferes with the appropriate processing of the SCI-57 insulin analog.

The peptide FVNQHLCGSDLVEALYLVCGER was identified on NLE-SCI-57 (**Supplementary Figure S6**). This reinforces the evidence that SCI-57 is functionally expressed in *N. benthamiana*.

Recombinant proteins expressed in plants usually maintain their native structural properties, facilitating recognition by antibodies. Specific recognition of SCI-57 was observed by two monoclonal antibodies, which were directed against separate antigenic determinants on the insulin molecule by ELISA. Although the ELISA test did not show a concentration similar to the Bradford method, results confirm that when SCI-57 is expressed without M2 and purified by FPLC, it is functionally active. Furthermore, most commercial insulin assays fail to detect recombinant insulin analogs (Heald et al., 2006); therefore, SCI-57 may tend to be underestimated.

As insulin and insulin analogs stimulate the development of adipose tissue (Hartman, 2008), we evaluated the effect of *Nb* preparations on the adipose differentiation of 3T3-L1 cells induced with an insulin-lacking adipogenic medium. Our results indicate that NLE-SCI-57 partially, and NLE-SCI-57/M2 advantageously, replace insulin’s role on 3T3 adipogenesis, whereas NLEN did not stimulate lipid accumulation in these cells. When the effect of *Nb* preparations on lipid accumulation was evaluated in the presence of insulin, both NLE-SCI-57 and NLE-SCI-57/M2 samples showed a synergic effect with the hormone stimulating more than twice the lipid accumulation in 3T3-L1 cells. As in the insulin-lacking condition, NLEN sample did not stimulate 3T3 lipid accumulation in the presence of the hormone (**Figure 10**). These results prove that our agroinfiltrated *Nb* preparations possess insulin-mimetic properties.

Hypoglycemic drugs exert their effects by any of the following three mechanisms: by diminishing intestinal glucose, increasing insulin secretion, or stimulating glucose uptake by insulin-targeted tissues such as adipose or skeletal muscle tissues. This last hypoglycemic mechanism is the most promising therapeutic target for both Type 1 and Type 2 diabetes.

In order to determine whether SCI-57 has an insulin-mimetic effect by stimulating the incorporation of glucose in adipocytes, we evaluated its effects on the uptake of 2-NBDG in 3T3-L1 adipocytes (**Figure 11**). NLE-SCI-57 promoted 2-NBDG incorporation by adipocytes almost at the same level as RGZ. In contrast, NLE-SCI-57/M2 exhibit a lower 2-NBDG incorporation than RGZ. We also evaluated the effect of purified fractions from agroinfiltrated *Nb* preparations on the incorporation of glucose. The purified NLE-SCI-57 fractions (**HPLC**, **Figure 11**) stimulates the incorporation of 2-NBDG to a greater extent than insulin; whereas purified NLE-SCI-57/M2 fractions (**A1–A3 and A4–A9**, **Figure 11**) are incorporated to a lesser extent than purified NLE-SCI-57 fractions. The high capacity of NLE-SCI-57 to stimulate the uptake of glucose in terminal adipocytes, and its low adipogenic capacity (pro-obesity), make it an optimal candidate for replacing native insulin. Whereas the NLE-SCI-57/M2 sample, has low (relative) incorporation of 2-NBDG and a marked pro-adipogenic effect (**Figure 10**).

Surprisingly, NLEN stimulated glucose uptake to the same extent as NLE-SCI-57/M2 (**Figure 11**), suggesting the *Nb* extract itself can incorporate glucose in 3T3-L1 adipocytes. Currently, we have no explanation for this effect. Although we and others have documented insulino-mimetic effects in diverse plant extracts (Naowaboot et al., 2012; Ortiz-Andrade et al., 2012; Khattak et al., 2013; Eddouks et al., 2014; Zapata-Bustos et al., 2014a), to the best of our knowledge, this property has not yet been evaluated for *Nicotiana* species.

Plants expressing heterologous proteins manifested at least 1.4 greater quantity of TSP than non-agroinfiltrated plants (**Figure 1C**). We conducted a proteomic analysis of the NLE of the different protein expression systems (GFP and SCI-57) using as control a NLEN to estimate the overall impacts on the biochemical pathways and protein synthesis in order to characterize the specific effects of these treatments at the cell-wide scale. However higher TSP content could be only related with pathogenesis and stress-related proteins produced as response of agroinfiltration (**Table 1**).

Targeted depletion pathogenesis-related and stress-inducible proteins may improve *N. benthamiana* as a protein expression platform and help identify the proteome changes in *N. benthamiana* when expressing different heterologous proteins with the aim to improve protein yield.

## Conclusions

For the first time, the results presented here show that *N. benthamiana* plants are capable of producing a biologically active insulin analog; SCI-57. The purification process enables us to extract pure SCI-57, in its active form, from a complex matrix of plant proteins, applying a time effective procedure. From the protein characterization experiments, we conclude that strategies to increase SCI-57 expression and accumulation may interfere with proper folding and the KDEL cleavage, generating the absence on disulfide bond formation and the KDEL peptide presence on the protein sequence. Even though SCI-57 in the lack of M2 co-expression produces a mixture of SCI-57 and SCI-57+KDEL, glycemic control was demonstrated through the 2-NBDG uptake by 3T3-L1 adipocytes, without any apparent pro-adipogenic or anti-adipogenic effects.

However, when SCI-57 is co-expressed with M2, it appears that M2 inhibits the cleavage of KDEL from SCI-57, and tends to encourage the formation of oligomeric forms, although further experiments are required to fully validate these findings.

When M2 is not co-expressed, the insulin analog SCI-57 displayed a lower pro-adipogenic effect and had a higher 2-NBDG uptake compared to when M2 is co-expressed. Overall our observations suggest that SCI-57 exerts its anti-diabetic properties, stimulating glucose uptake, without affecting the development of adipose tissue.

Proteome changes related to the expression of heterologous proteins on *N. benthamiana* were not observed; up-regulated proteins were related to the agroinfiltration process. However, further experiments are required, employing leaves agroinfiltrated with an empty vector.

## Author Contributions

AM-T: Conceived of this idea, carried out the experiments, processed the experimental data, performed the analysis, drafted the manuscript, and designed the figures. JR and KL: Label-free proteomic analysis experimental design, assessment of the structural analyses by diagonal electrophoresis. MI-C: Contributed to the interpretation of the results and English revision, final approval of the version for submission. LS-O: Conceived, planned, financed the adipocyte experiments, and provided a critical review of the final manuscript. AE-M: Design and technical execution of MS analysis, interpretation of analytical assays, A critical review of the final manuscript. AL-C: Technical support for molecular cloning, transient expression in *N. benthamiana* and qRT-PCR for gene expression analysis, data acquisition and revision of the manuscript. MG-L: Key role in the study design, editing the manuscript and critical revision for important intellectual content, a critical review of the final manuscript. AR-S: Study design and concept, financial support for part of the study, drafting and editing the manuscript and critical revision for important intellectual content, final approval of the version for submission. All authors discussed the results and commented on the manuscript.

## Funding

This work was supported in part by PROINPEP 2015, 2016 (AR-S), awarded to support Ph.D. students of the pharmacology program PROSNI 2016, 2017 to AR-S, and by own resources of the Doctors MG-L, LS-O, JR, and AR-S.

## Conflict of Interest

The authors declare that this research was conducted in the absence of any commercial or financial relationships that could be construed as a potential conflict of interest.
