# Supplementary material for: Expression of the Biologically Active Insulin Analog SCI-57 in Nicotiana Benthamiana
Source: Front Pharmacol. 2019 Nov 14;10:1335. doi: 10.3389/fphar.2019.01335 (PMC6868099; doi:10.3389/fphar.2019.01335)
Supplement: Supplementary file 1 [file Table_1.docx]

# Supplementary Material

**Supplementary Table S1**. The sequence of primers for RT-PCR and qRT-PCR.

| Primer | |  | | Forward (5´-3´) | Reverse (5´-3´) |
| --- | --- | --- | --- | --- | --- |
| 18 S |  | | cggcggatgttgcttttagg | | gacctggtaagtttccccgtg |
| GFP |  | | ggtgagcaagggcgaggagct | | gcttgccggtggtgcagatgaact |
| SCI-57 |  | | tcgccggaaagcagttgg | | cctcccccggtcttgtcg |

**Supplementary Table S2.**  Proteins^1^ up-regulated by at least twofold in plants expressing heterologous proteins compared to non-infiltrated control leaves.

| **NCBI accession** | **Protein name** | **Relative abundance (n-fold)** | | | |
| --- | --- | --- | --- | --- | --- |
|  |  | $\frac{NLE GFP}{\mathrm{NLEN}}$ | $\frac{NLE SCI-57}{\mathrm{NLEN}}$ | $\frac{NLE SCI-57/M2}{\mathrm{NLEN}}$ | |
| XP_009760305.1 | glucan endo-1,3-beta-glucosidase, basic vacuolar isoform isoform X2 | 56.6 | 15.4 | 19.7 | |
| XP_009593782.1 | peroxidase P7-like | 31.1 | 8.7 | 24.0 | |
| XP_019265981.1 | alpha-galactosidase 1-like | 2.7 | 4.4 | 2.8 | |
| XP_019232131.1 | acid beta-fructofuranosidase AIV-18-like | 3.4 | 9.4 | 2.2 | |
| XP_009760245.1 | alpha-galactosidase | 2.6 | 3.8 | 3.4 | |
| AAA34078.1 | beta(1,3)-glucanase regulator | 32.5 | 34.5 | 37.3 | |
| NP_001312082.1 | peroxidase N1 precursor | 8.9 | 2.3 | 6.6 | |
| ANW86204.1 | beta-hexosaminidase 1 | 4.4 | 2.7 | 2.4 | |
| XP_019232207.1 | probable inactive purple acid phosphatase 27 | 5.2 | 4.8 | 5.3 | |
| XP_009606552.1 | pectinesterase/pectinesterase inhibitor U1 | 2.3 | 4.0 | 2.5 | |
| XP_016511304.1 | acetylajmalan esterase-like | 3.1 | 9.4 | 2.3 |  |
| XP_019224644.1 | pectinesterase-like | 6.0 | 4.9 | 5.2 |  |
| XP_009617302.1 | endochitinase A | 69.5 | 37.6 | 36.2 | |
| XP_019225618.1 | pathogenesis-related protein R major form | 6.8 | 6.3 | 7.1 | |
| XP_009771097.1 | probable ribose-5-phosphate isomerase 3, chloroplastic | 22.6 | 19.6 | 2.1 | |
| CBK52316.1 | Nb cell deth marker | 9.3 | 13.3 | 15.2 | |
| XP_009782398.1 | osmotin | 34.2 | 2.4 | 16.2 | |
| XP_019238089.1 | basic form of pathogenesis-related protein 1 | 27.7 | 5.8 | 43.6 | |
| XP_019244706.1 | kunitz trypsin inhibitor 2-like | 72.4 | 50.5 | 53.1 | |
| XP_019261786.1 | wound-induced protein WIN1 | 31.7 | 7.4 | 49.1 | |
| XP_009766291.1 | lignin-forming anionic peroxidase-like | 6.0 | 4.2 | 7.8 | |
| XP_009768114.1 | expansin-like A2 | 7.7 | 13.8 | 9.1 | |
| XP_018623099.1 | receptor-like protein 12 | 2.1 | 6.0 | 2.4 | |
| XP_016478788.1 | cysteine-rich repeat secretory protein 38-like | 18.5 | 4.4 | 7.3 | |
| XP_019226991.1 | heme-binding protein 2 | 14.1 | 5.0 | 2.9 | |
| NP_001312988.1 | uncharacterized protein LOC107820113: Plant basic secretory protein (BSP) family protein | 36.7 | 194.6 | 172.4 | |
| XP_019255926.1 | uncharacterized protein LOC109234402: Transmembrane protein | 10.1 | 24.0 | 20.6 | |
| XP_009781670.1 | pathogenesis-related protein R minor form | 15.6 | 18.8 | 15.5 | |
| XP_009763689.1 | pathogenesis-related protein PR-4A | 8.5 | 4.6 | 23.8 | |
| OIT28720.1 | basic endochitinase | 23.2 | 10.6 | 6.7 | |
| XP_016504046.1 | serine carboxypeptidase-like 20 | 3.9 | 2.4 | 2.9 | |
| XP_019224145.1 | low-temperature-induced cysteine proteinase-like | 4.2 | 2.7 | 2.1 | |
| XP_019249750.1 | uncharacterized protein LOC109228938: Transmembrane protein | 6.4 | 6.6 | 2.4 | |
| XP_019249072.1 | uncharacterized protein At5g01610-like : Pectinesterase | 5.9 | 9.6 | 6.6 | |
| XP_019265536.1 | non-specific lipid transfer protein GPI-anchored 1 | 15.1 | 15.5 | 9.7 | |
| XP_019241188.1 | aquaporin PIP-type pTOM75 | 13.3 | 20.3 | 6.6 | |
| XP_016495116.1 | suberization-associated anionic peroxidase-like | 58.7 | 8.8 | 64.5 | |
| XP_009762395.1 | pectinesterase/pectinesterase inhibitor U1 | 2.1 | 6.2 | 2.7 | |
| XP_015951897.1 | ferredoxin--NADP reductase, leaf isozyme, chloroplastic | 6.9 | 3.8 | 3.4 | |
| XP_004234419.1 | peroxidase P7-like | 8.4 | 4.0 | 11.4 | |
| XP_009787159.1 | basic form of pathogenesis-related protein 1-like | 313.3 | 81.0 | 128.3 | |
| XP_019703249.1 | pectinesterase-like | 26.4 | 28.4 | 6.0 | |
| XP_002312923.2 | Chain A family protein | 6.9 | 3.9 | 15.4 | |
| NP_001167809.1 | uncharacterized protein LOC100381507 precursor : Peroxidase P7 | 15.7 | 42.3 | 31.6 | |
| OVA02043.1 | Uncharacterized protein family : Plant basic secretory protein | 46.2 | 189.3 | 397.9 | |
| prf\|\|1202235A | protein 1a,pathogenesis related | 22.1 | 35.2 | 10.9 | |
| XP_022145284.1 | thaumatin-like protein 1b | 244.5 | 15.0 | 339.6 | |
| XP_021284600.1 | zingipain-2 | 7.9 | 10.8 | 4.2 | |

^1^Proteins were listed base on the n-fold expression normalized with NLEN. Ratios were inferred from MS/MS peptide abundance values determined for each protein.

**Supplementary Table S3.** Proteins^1^ down-regulated by at least twofold in plants expressing heterologous proteins compared to non-infiltrated control leaves.

| **NCBI accesion** | **Protein name** | **Relative abundance (n-fold)** | | |
| --- | --- | --- | --- | --- |
|  |  | $\frac{NLE GFP}{\mathrm{NLEN}}$ | $\frac{NLE SCI-57}{\mathrm{NLEN}}$ | $\frac{NLE SCI-57/M2}{\mathrm{NLEN}}$ |
| XP_019267732.1 | putative lactoylglutathione lyase | 0.13418922 | 0.16042636 | 0.30238739 |
| XP_019246895.1 | uncharacterized protein At4g14100-like: Transferring glycosyl group transferase | 0.05508274 | 0.08592561 | 0.09234485 |
| XP_019265330.1 | pectin acetylesterase 12-like | 0.10000416 | 0.15565843 | 0.33595339 |
| XP_009608768.1 | subtilisin-like protease SBT1.2 | 0.23836271 | 0.03142879 | 0.15891894 |
| XP_019262089.1 | uncharacterized protein LOC109239937: Thioredoxin superfamily protein | 0.38297814 | 0.32583563 | 0.36519268 |
| XP_009791746.1 | oxygen-evolving enhancer protein 3-2, chloroplastic-like | 0.00226326 | 0.00165505 | 0.01010628 |
| XP_016466644.1 | subtilisin-like protease SBT1.9 | 0.38517833 | 0.17236907 | 0.40224054 |
| XP_009624033.1 | cucumber peeling cupredoxin-like | 0.1612344 | 0.09862287 | 0.17893193 |
| XP_009800887.1 | non-specific lipid-transfer protein 1-like | 0.46392661 | 0.23525174 | 0.48393808 |
| XP_019228947.1 | lysM domain-containing GPI-anchored protein 1-like | 0.15016731 | 0.23838725 | 0.46358422 |
| XP_015168029.1 | probable polygalacturonase isoform X1 | 0.03106154 | 0.11347166 | 0.17646495 |
| XP_019230322.1 | cysteine-rich receptor-like protein kinase 2 | 0.22151981 | 0.09507234 | 0.2323628 |
| XP_019253164.1 | aspartyl protease AED3-like | 0.16188079 | 0.07515577 | 0.13197053 |
| XP_009769844.1 | defensin-like protein P322 | 0.38556323 | 0.0525195 | 0.46658465 |
| XP_019248145.1 | DNA-damage-repair/toleration protein DRT100-like | 0.09598252 | 0.07322619 | 0.26816767 |
| XP_019261049.1 | cytochrome b561 and DOMON domain-containing protein At5g47530-like | 0.19842684 | 0.03909963 | 0.30771869 |
| XP_015055513.1 | 5-methyltetrahydropteroyltriglutamate--homocysteine methyltransferase | 0.25078616 | 0.16219038 | 0.45525193 |
| XP_004143760.1 | alpha-mannosidase | 0.1496509 | 0.19319926 | 0.38126675 |
| XP_016580306.1 | auxin-binding protein ABP19a-like | 0.00778327 | 0.44839232 | 0.46250882 |
| XP_016540540.1 | leucine-rich repeat extensin-like protein 4 | 0.08594779 | 0.05400536 | 0.36935251 |
| XP_021892086.1 | polygalacturonase | 0.26879667 | 0.08103308 | 0.28860269 |
| XP_019263400.1 | thioredoxin F-type, chloroplastic-like | 0.47818338 | 0.36085941 | 0.45711738 |
| XP_009796710.1 | 21 kDa protein-like | 0.1985814 | 0.10829054 | 0.19501423 |

^1^Proteins were listed base on the n-fold expression normalized with NLEN. Ratios were inferred from MS/MS peptide abundance values determined for each protein.

**Supplementary Figures**

**Supplementary Figure S1**


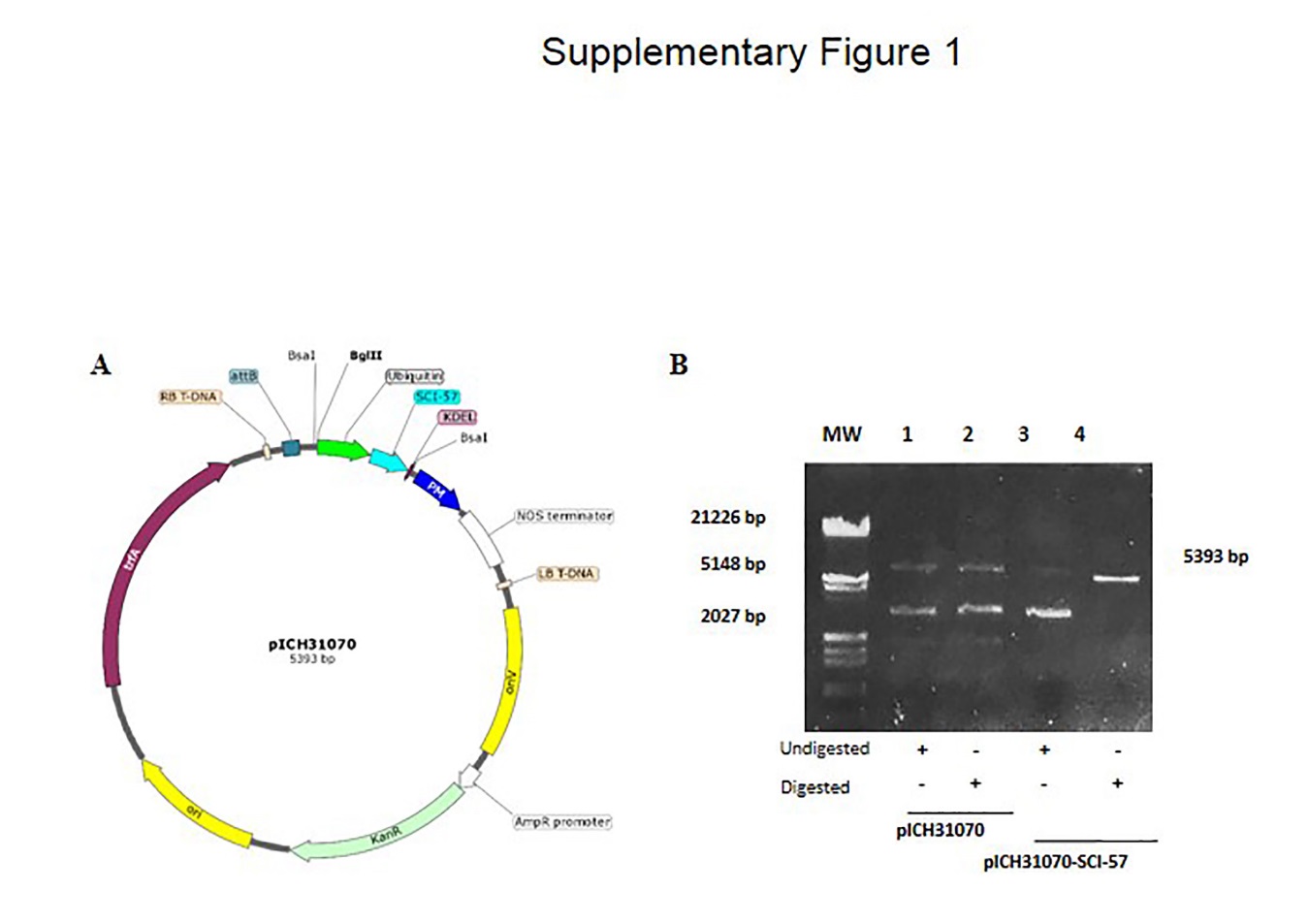


**pICH31070-SCI-57**

**Supplementary Figure S1.** A) Genetic map of the expression vector pICH31070 which contains the gene coding for SCI-57 (pICH31070 + SCI-57) indicating the digestion site with the enzyme *Bgl*II. The genetic map was generated by the SnapGene program (GSL Biotech LLC, Chicago, IL, USA). B) Restriction mapping of the assembled expression vector. Mw: Molecular weight marker, 1: undigested pICH31070, 2: pICH31070 digested, 3: pICH31070-SCI-57 undigested and 4: pICH31070- SCI-57 digested. RB T-DNA: Righ border transfer DNA, AttB: site-specific recombinase, *Bsa*I: *Bsa*I enzyme recognition site, *Bgl*II: *Bgl*II enzyme recognition site, PM: Partial movement protein, NOS terminator: Termination sequence of the nopaline synthase gene, LB T-DNA: Left border transfer DNA, oriV: Replication Origin, Ampr promoter: Transcriptional regulator, KanR: Kanamycin resistance, trfA: replication protein that binds and activates oriV.

**Supplementary Figure S2**


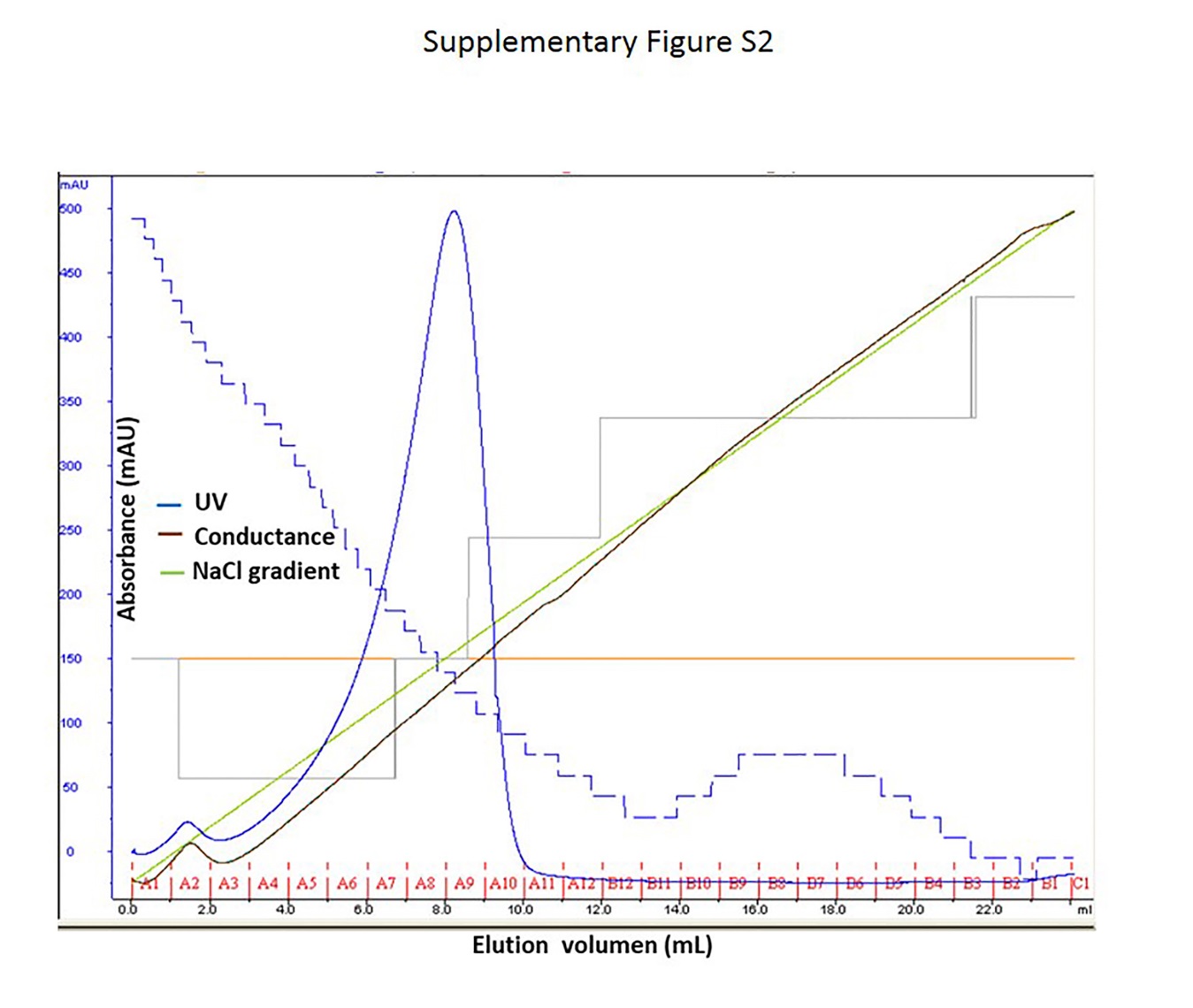


**Supplementary Figure S2.** Insulin cation exchange separation performed on SP-Sepharose Fast Flow. Five ml of the sample was loaded (1.75 mg/ml). Elution fractions were 1 ml, and the flow rate was set at 0.4 ml/min 100 mM citrate buffer pH 4.3 (elution with 0-1 M linear NaCl gradient). Absorbance was expressed in milli-absorbance units for the A280 (blue). Fractions were labeled by the A-B letters from 1-12 numbers.

**Supplementary Figure S3**


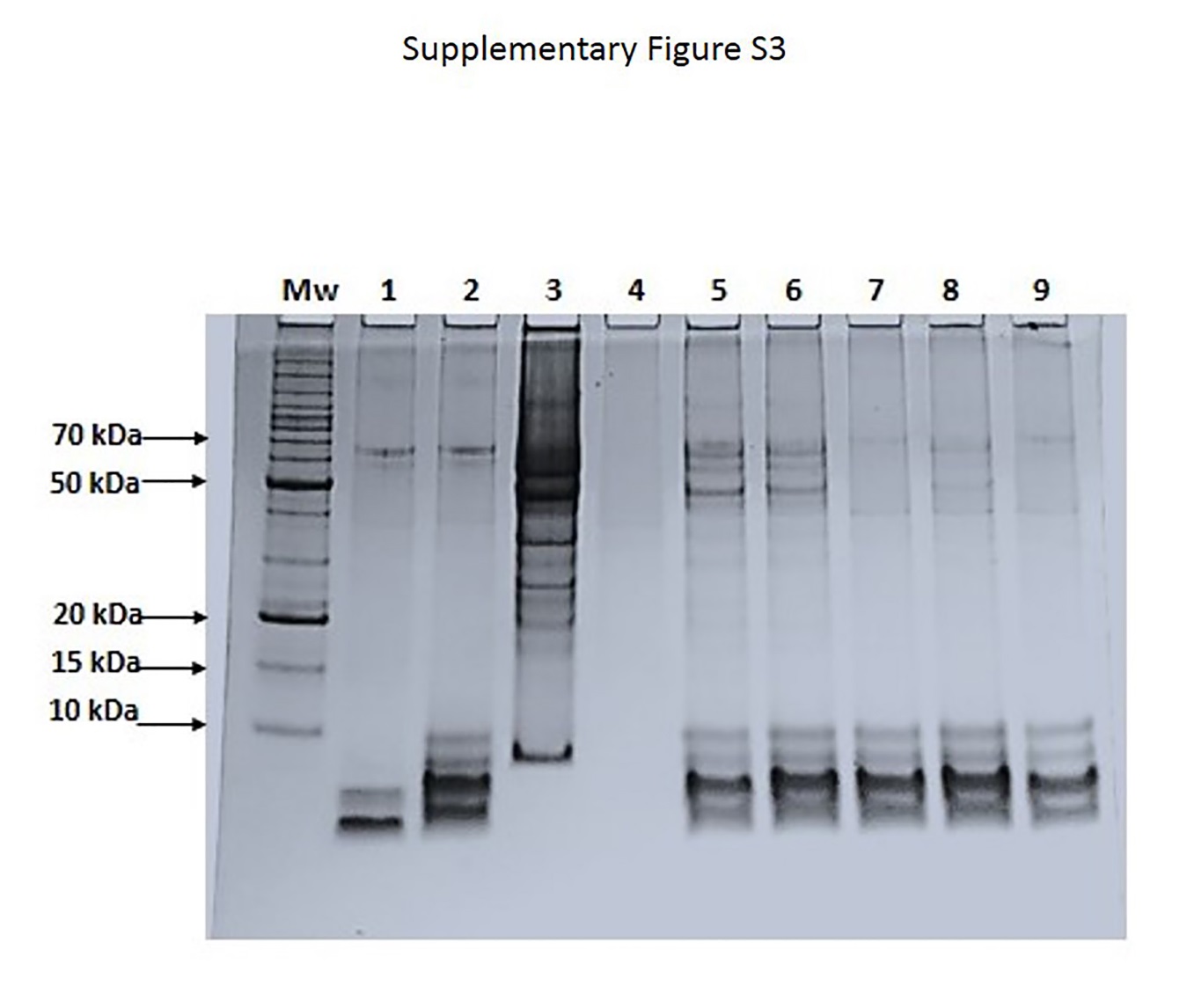


**Supplementary Figure S3.** SDS-PAGE gel- 13% Glycine stained with Coomassie blue to analyze the fractions recovered from the purification of recombinant human insulin (RHI). Lane 1: 100 U / ml RHI (PISA pharmaceutical, México) 3.5 μg; Lane 2: Sample prepared from 100 U / ml RHI (PISA pharmaceutical, México) in a concentration of 1.75 mg/ml diluted in 100 mM citrate buffer pH 4.3; lane 3: NLE-SCI-57. From lane 5 to 9, fraction A7 to A11 respectively from the insulin purification by cationic exchange.

**Supplementary Figure S4**


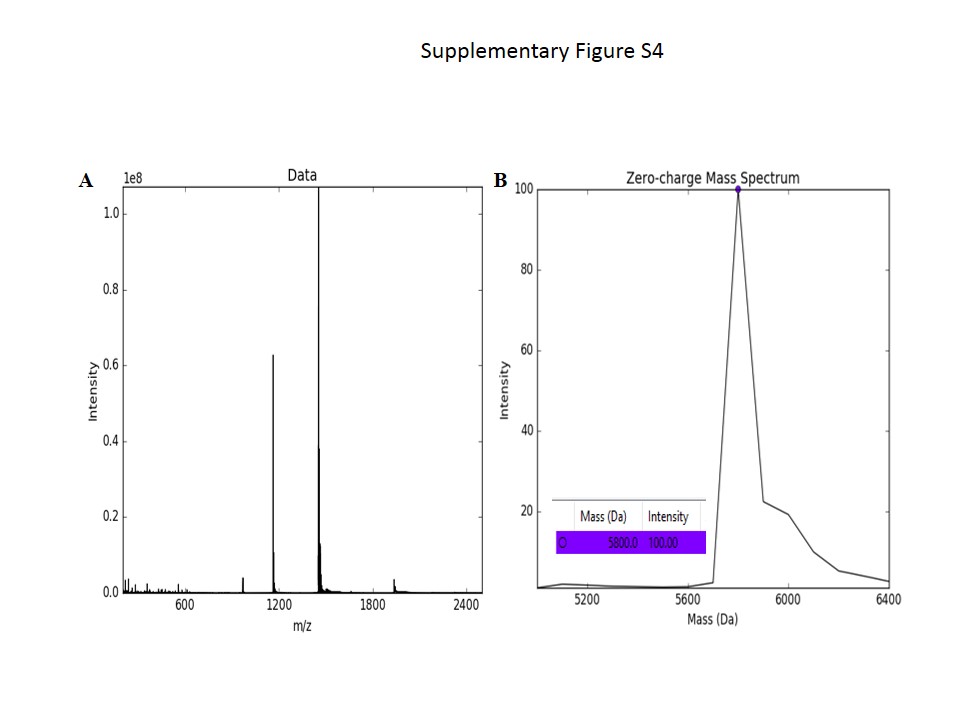


**Supplementary Figure S4**. MS spectra analysis of native insulin. A) The molecular weight of insulin by applying deconvolution to the spectrum obtained by MS. B) Spectrum obtained by MS of the EP 30 μM insulin sample.

**Supplementary Figure S5**


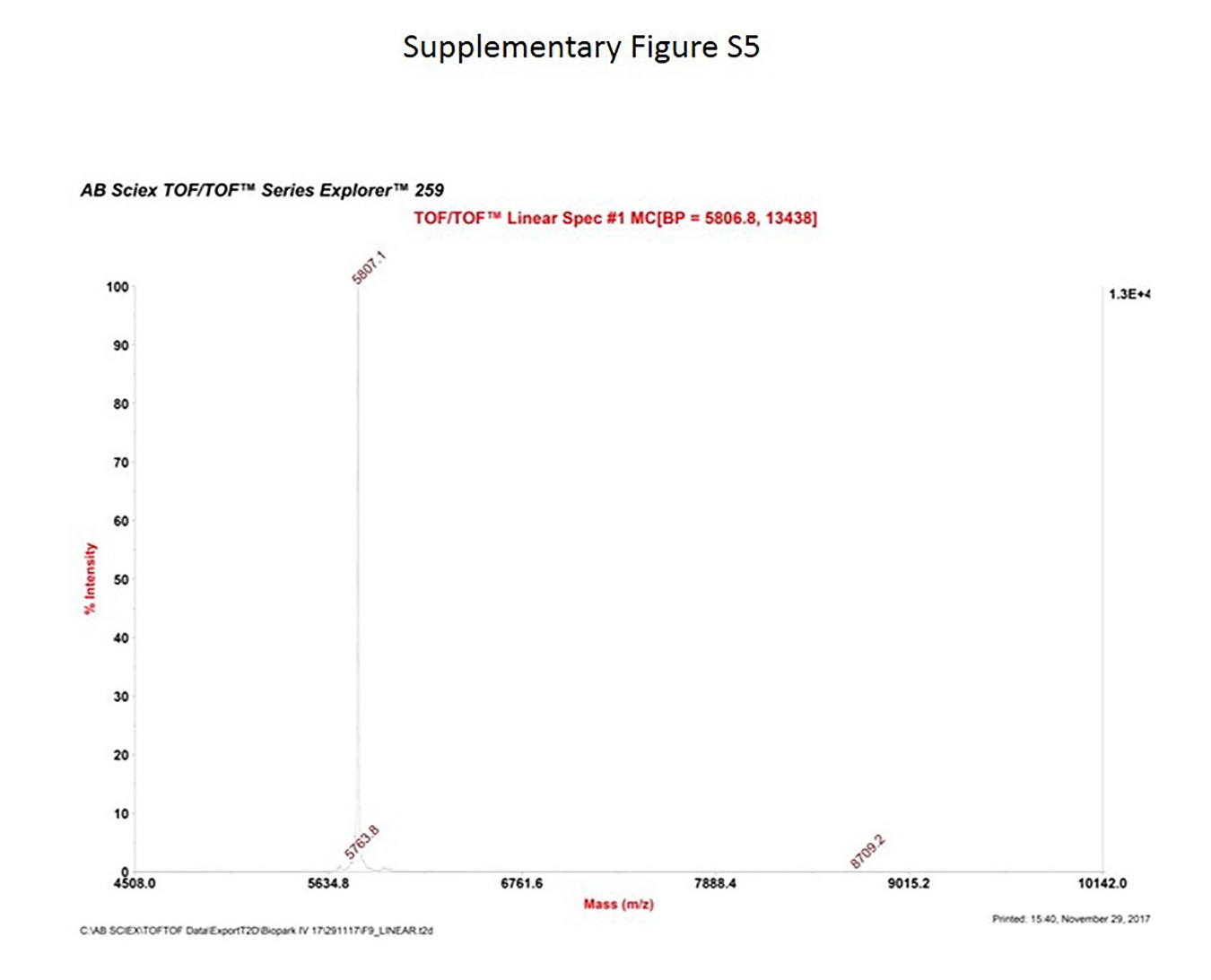


**Supplementary Figure S5.** Mass spectrum of the insulin sample obtained using MALDI TOF / TOF.

**Supplementary Figure S6**


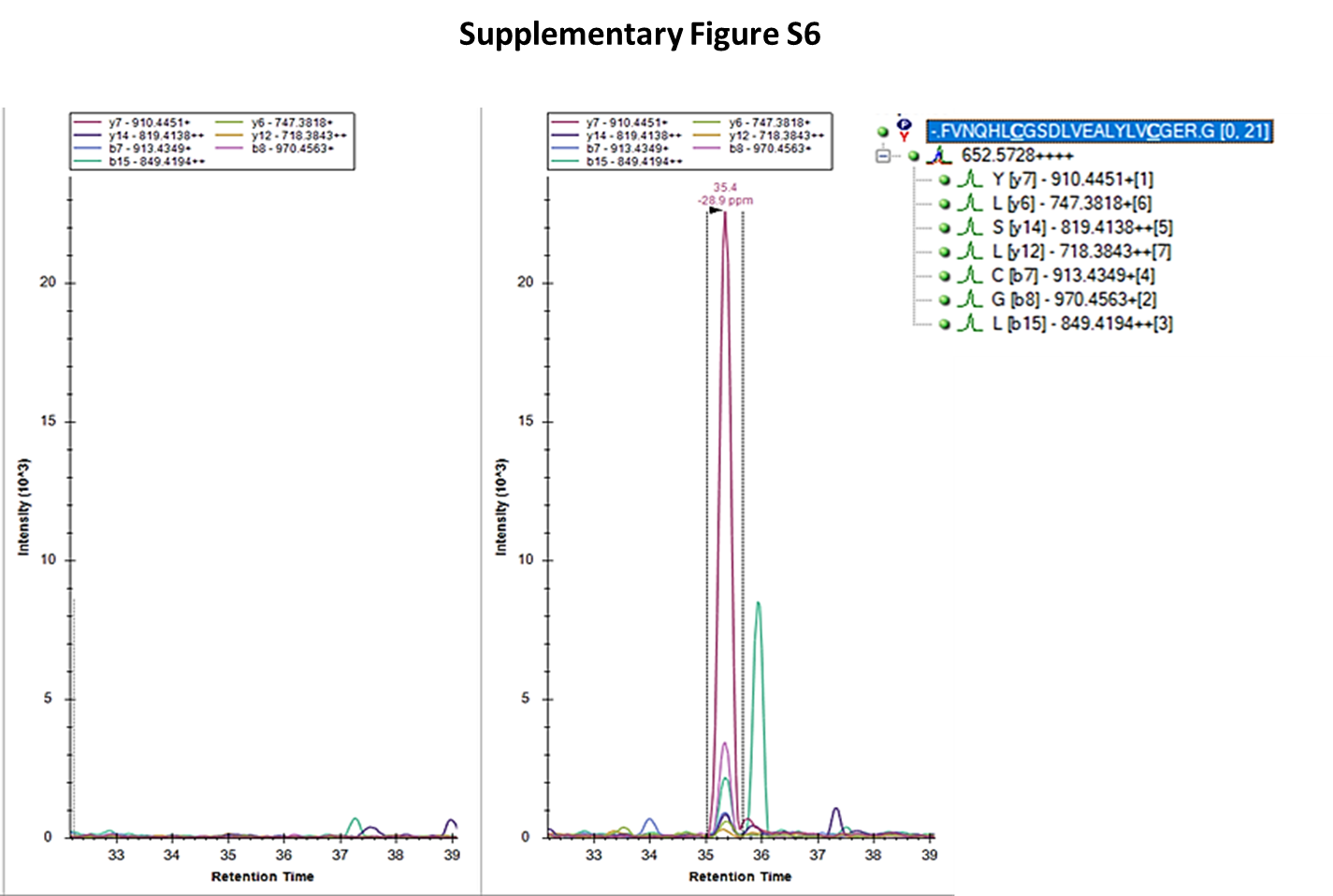


**Supplementary Figure S6.** MS-MS spectra of the peptide FVNQHLCGSDLVEALYLVCERG showing extracted fragment ion chromatograms for the seven most intensive peptide fragments. The image on the left corresponds to the NLEN sample and the image on the right to NLE SCI-57 sample.
